# Comparison of volatile compounds in different parts of fresh *Amomum villosum* Lour. from different geographical areas using cryogenic grinding combined HS–SPME–GC–MS

**DOI:** 10.1186/s13020-020-00377-z

**Published:** 2020-09-07

**Authors:** Ling-Xiao Chen, Yun-Feng Lai, Wei-Xiong Zhang, Jing Cai, Hao Hu, Ying Wang, Jing Zhao, Shao-Ping Li

**Affiliations:** 1 State Key Laboratory of Quality Research in Chinese Medicine, Institute of Chinese Medical Sciences, University of Macau, Zhuhai, Macao SAR China; 2 Center for Ecological and Environmental Science, Northwestern Polytechnical University, Xi’an, 710072 China

**Keywords:** Fresh *Amomum villosum* Lour., HS–SPME–GC–MS, Volatile compounds, Cryogenic grinding

## Abstract

**Background:**

The essential oil is one of the main active ingredients of *Amomum villosum* Lour. However, volatile compounds are easily lost during the drying, storage and even sample preparation procedure. Therefore, using fresh samples can obtain more accurately data for qualitative and comparative analysis.

**Methods:**

In this study, the volatile compounds in different parts of fresh *A. villosum* from different origins were systemic analyzed and compared by using cryogenic grinding combined HS–SPME–GC–MS for the first time. GC–MS analyses were performed on a 6890 Series GC instrument coupled to a 5973 N mass spectrometer. The volatile compounds were extracted by the SPME fiber (100 μm PDMS). Analytes separation was achieved on a HP-5MS capillary column. The oven temperature was initially programmed at 70 °C, then raised 4 °C/min to reach 125 °C and then programmed at 0.5 °C/min to 133 °C, then at 6 °C/min to 170 °C and finally, at 20 °C/min to 280 °C held for 2 min. The temperatures of the injection port, ion source and transfer line were set at 250 °C, 230 °C and 280 °C, respectively.

**Results:**

Forty-eight main compounds were identified in different parts of fresh *A. villosum*. The most abundant components in fresh fruit samples were camphor (3.91%), bornyl acetate (10.53%), caryophyllene (8.70%), β-bisabolene (11.50%), (E)-nerolidol (14.82%) and cubenol (10.04%). This is quite different with that of dried samples analyzed in our previous work. As different parts of the same plant, many common components with biological activities were detected in fruit and other parts. In principle components analysis (PCA) and hierarchical clustering analysis (HCA), four parts of *A. villosum* were divided into different groups clearly. Additionally, fruit and root samples also could be divided into two subgroups (HCA) in accordance with their regions.

**Conclusion:**

The developed method was successfully used for qualitative and comparative analysis of volatile compounds in fresh *A. villosum* samples. Additionally, using fresh samples can obtain much more information which is helpful for their performance in the fields of functional foods, agriculture and biomedical industry. Furthermore, our research is helpful for comprehensive utilization and quality control of *A. villosum*.

## Introduction

*Amomum villosum* Lour., belongs to the Zingiberaceae family, is wildly cultivated in southern China, especially in Yangchun, Guangdong province (111° 16′ 27″ to 112° 09′ 22″ E, 21° 50′ 36″ to 22° 41′ 01″ N) [1, 2]. The dried ripe fruit of *A. villosum*, also named Amomi Fructus, has been used as traditional medicine to treat gastritis, stomachache, and digestive diseases for hundreds of years [3]. Recently, Amomi Fructus is also authorized as a food by the China Food and Drug Administration [4]. As the main active ingredient in Amomi Fructus, essential oil possesses many biological activities such as antimicrobial, anti-inflammation and analgesic effects [5–7]. Due to the excellent functions and special aroma of its oil, Amomi Fructus presents great economic values in the functional foods and biomedical industry. Nowadays, it has been used as natural raw materials for wine, tea, candies and cosmetics. Additionally, Amomi Fructus is also one of the food spices in China [8]. With the development of Amomi Fructus related industry, the natural resource of Amomi Fructus is hard to meet the increasing demand [1, 9]. Generally, different parts of the plant have similar secondary metabolites. Expand the application parts are helpful for making up the shortages of this resource. Therefore, qualitative and comparative analysis of the essential oil in different parts of *A. villosum* from different geographical areas is very necessary, which is beneficial for comprehensive utilization of this these resources as well as quality control of *A. villosum*.

The chemical composition of essential oil in *A. villosum* has been reported by several papers [10–14]. However, due to the fresh *A. villosum* samples is difficult to collect and store, most of these studies were focused on dried samples. Actually, volatile compounds are easily lost during the drying, storage and even sample preparation procedure. Therefore, using fresh samples can obtain more accurately data for qualitative and comparative analysis. Conventional techniques such as steam distillation and supercritical CO_2_ extraction coupled with gas chromatography–mass spectrometry (GC–MS) have been employed to investigate the essential oils in fresh plants [15–18]. However, these techniques either need a tedious and time-consuming sample preparation procedure or require special and expensive equipment. Headspace solid-phase microextraction (HS-SPME) is a convenient alternative to traditional essential oil extraction methods. It allows extraction and simultaneous concentration of analytes in one single step. Furthermore, no organic solvents were needed in the whole extraction process [19, 20]. In recent years, HS-SPME coupled GC-MS has gained wide applications in volatile compounds analysis especially in case on natural plants [21–24].

The sample preparation methods for HS–SPME–GC–MS analysis of herbs usually contain pulping, milling and so on. However, the frictional forces arising during the milling process result in the temperature increasing which is enhancing the losses of essential oil and thermo-sensitive components through evaporation and oxidative reactions [25]. As the literature report, the losses of essential oil in nutmeg and cinnamon are about 37% and 17%, respectively [26]. Therefore, a volatile compounds saver sample preparation method is very necessary. Cryogenic grinding technique using liquid nitrogen that provides extremely low temperature needed to pre-cool the sample. During this process, the moisture and oil are solidified so that the sample becomes brittle. Additionally, to maintain the low temperature, the liquid nitrogen vaporized to gaseous state and creates an inert and dry atmosphere which further protects the sample [27, 28]. As the literature report, cryogenic grinding obtained 29.5% more volatile oil in cloves than that of ambient grinding [28].

In this work, the volatile compounds in 72 fresh *A. villosum* samples were analyzed and compared by using cryogenic grinding combined HS–SPME–GC–MS. As far as we are aware, this is the first time for systemic qualitative and comparative analysis of volatile compounds in different parts of fresh *A. villosum* from different geographical areas. Additionally, this work is helpful for comprehensive utilization and quality control of this plant.

## Materials and methods

### Materials and chemicals

Yangchun is located in southwest of Guangdong province of China (111° 16′ 27″ to 112° 09′ 22″ E, 21° 50′ 36″ to 22° 41′ 01″ N), which is the geo-authentic habitats of *A. villosum.* Seventy-two batches of fresh *A. villosum* samples containing fruits (1–18), roots (19–36), leaves (37–54) and stems (55–72) were obtained from different places of Yangchun. The detailed characteristics are presented in Table 1. Species identification was performed by Dr. Jing Zhao and Dr. Hao Hu. The voucher specimens of these samples were deposited at the Institute of Chinese Medical Sciences, University of Macau, Macao SAR, China.

**Table 1:** Characteristics of analyzed samples

Codes	Sample	Source
1–9	Fruits	Pingxi village, Yangchun
10–18	Fruits	Shiwanzai village, Yangchun
19–27	Roots	Pingxi village, Yangchun
28–36	Roots	Shiwanzai village, Yangchun
37–45	Leaves	Pingxi village, Yangchun
46–54	Leaves	Shiwanzai village, Yangchun
55–63	Stems	Pingxi village, Yangchun
64–72	stems	Shiwanzai village, Yangchun

All other chemicals and reagents were of analytical grade. The extraction fibers: 100 μm polydimethylsiloxane (PDMS), 65 μm polydimethylsiloxane/divinylbenzene (PDMS/DVB), 85 μm polyacrylate and 75 μm carboxen poly(dimethylsiloxane) (CAR/PDMS) were purchased from Supelco (Bellefonte, PA, USA). Headspace vials (20 mL) and accessories were obtained from Agilent (Palo Alto, CA, USA).

### Experimental procedures for cryogenic grinding of fresh *A. villosum* samples

In order to reduce the loss of volatile compounds in the sample preparation procedure, cryogenic grinding was employed. Fresh *A. villosum* samples (5 g) were frozen in liquid nitrogen, then volatilization of the liquid nitrogen and quickly crushed the sample in airtight grinding bowl. The procedure was repeated until the sample milling to fine powder (20 mesh). Then 50 mg sample powder was transferred to a 20 mL headspace vials for SPME analysis.

### Instrumentation and GC–MS conditions

The incubation, equilibrium, extraction and desorption of volatile components were carried out automatically by a Combi-Pal autosampler (CTC Analytics, Zwingen, Switzerland). Specifically, the headspace vials were incubated and equilibrated at 80 °C for 10 min, under continuous agitation (500 rpm). And then the volatile compounds were extracted by the SPME fiber (100 μm PDMS) for 30 min at the same condition. The desorption was performed in the injector at 250 °C for 5 min.

GC–MS analyses were performed on a 6890 Series GC instrument coupled to a 5973 N mass spectrometer and a ChemStation software (Agilent Technologies, Palo Alto, CA). Analytes separation was achieved on a HP-5MS capillary column (30 m × 0.25 mm i.d.) coated with 0.25 μm film (5% phenyl methyl siloxane). The splitless injection mode was used. High-purity helium was used as carrier gas with flow rate of 1 mL/min. The oven temperature was initially programmed at 70 °C, then raised 4 °C/min to reach 125 °C and then programmed at 0.5 °C/min to 133 °C, then at 6 °C/min to 170 °C and finally, at 20 °C/min to 280 °C held for 2 min. The temperatures of the injection port, ion source and transfer line were set at 250 °C, 230 °C and 280 °C, respectively. The mass spectrometer was operated in electron-impact mode (EI).

### Statistical data analysis

The principal components analysis (PCA) and hierarchical clustering analysis (HCA) analyses were performed in software R for windows based on the 72 *A. villosum* samples with 48 identified volatile compounds and their Comparative index. Specifically, the PCA was conducted using R package, gmodels. And the analysis results were visualized using R packages, ‘ggplot2’ and ‘scatterplot3d’. The heatmap analysis and HCA were performed using R package, ‘pheatmap’.

## Results and discussion

### Optimization of the SPME conditions

**Fig. 1:**
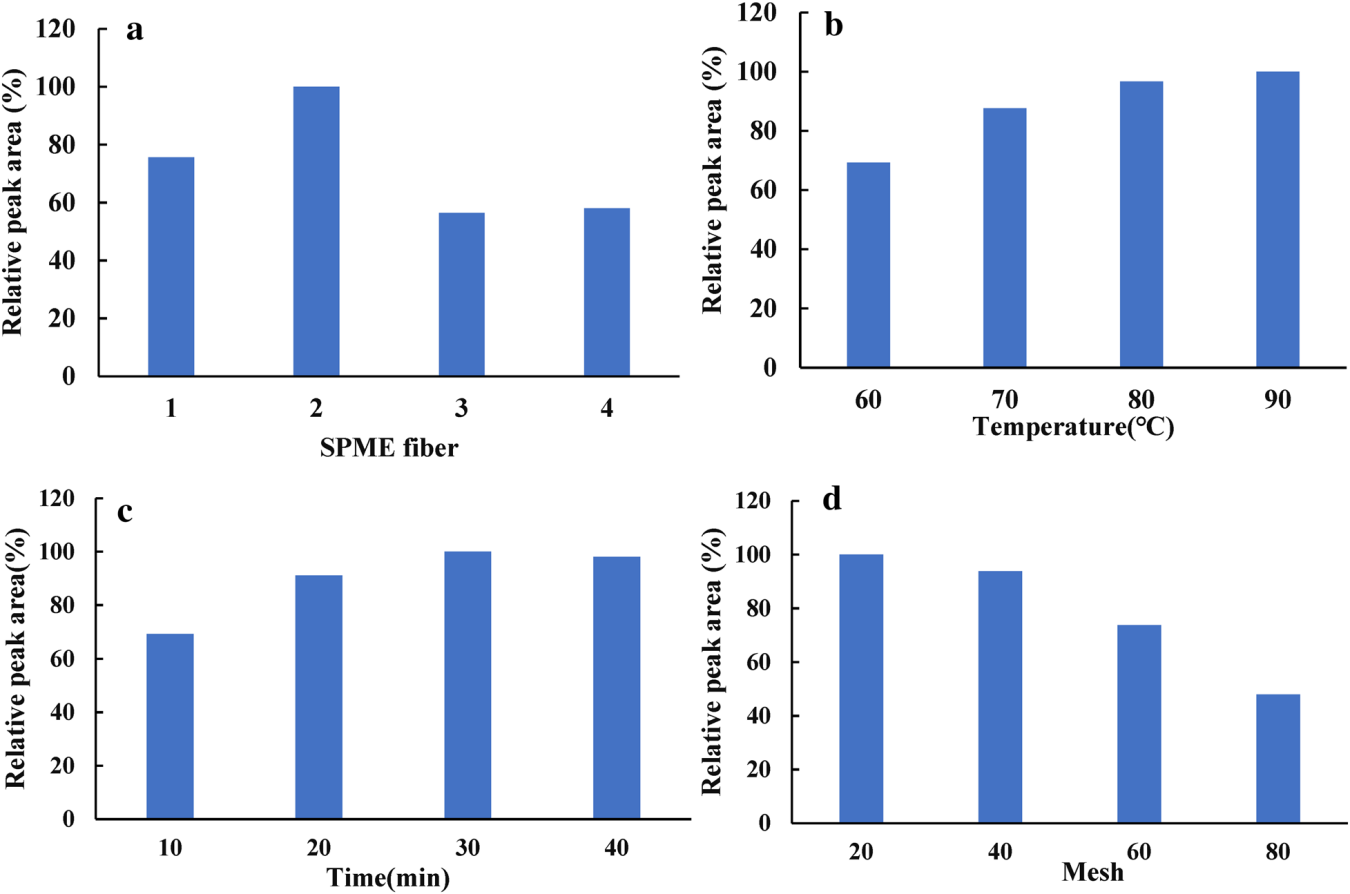
Optimization of SPME conditions. **a** SPME fiber (1, 65 μm PDMS/DVB; 2, 100 μm PDMS; 3, 85 μm Polyacrylate; 4, 75 μm CAR/PDMS). **b** Extraction temperature. **c** Extraction time. **d** Particle sizes. All data was presented as average of two determinations, their relative average deviations were less than 4.7%

For volatile compounds analysis, selected the appropriate SPME fibers is the first step. In this study, 65 μm PDMS/DVB, 100 μm PDMS, 85 μm polyacrylate and 75 μm CAR/PDMS were used to evaluate the effect of fiber types on the extraction of volatile compounds in fresh *A. villosum.* (Sample 1). The relative peak area (RPA) was employed to evaluate the content of volatile compounds under different SPME conditions. The RPA was calculated as: RPA (B) % = (B/A) *100%; (A was the total peak area of volatile compounds under the optimum condition; B was the total peak area of volatile compounds under other conditions) [29]. As shown in Fig. 1a, 100 μm PDMS achieved the highest extraction of the volatile compounds than other fibers. This indicated that the retention ability of this fiber for the volatile compounds in fresh *A. villosum* is much stronger than the other fibers. Additionally, the 65 μm PDMS/DVB absorbed more volatile compounds than 85 μm polyacrylate and 75 μm CAR/PDMS. Actually, the 100 μm PDMS, 65 μm PDMS/DVB are non-polar fiber and semi-polar fiber, respectively. Otherwise, 85 μm polyacrylate and 75 μm CAR/PDMS are polar fibers. The main volatile compounds in *A. villosum* such as bornyl acetate, borneol and camphor are low-polar compounds which are effective absorbed by the non-polar fiber or semi-polar fiber [30]. In addition, the fiber coating thickness also affects the extraction process. Usually, the small molecules are absorbed more efficiently by a thicker fiber [30]. The extraction temperature (60, 70, 80, 90 °C) at four levels were investigated. The results (Fig. 1b) showed that the content of volatile compounds increased steadily with temperature (60–80 °C). Actually, the extraction temperature had a significant influence on the adsorption process because it can influence the distribution coefficients of the volatile compounds among sample, headspace and fiber. When the temperature was higher than 80 °C, the content of volatile compounds was stable. However, higher temperature may also cause compound degradation. Therefore 80 °C was selected as the optimum temperature. The extraction time ranges from 10 to 40 min was investigated (Fig. 1c). When the extraction time increased from 10 to 30 min, the total content of volatile compounds was increased correspondingly. When the extraction time was higher than 30 min, the total content of volatile compounds was stable. Therefore 30 min was selected as the optimum extraction time. Particle size is also an important factor affecting the extraction efficiency. Usually, small particle size is easier for essential oil volatilize. However, conventional sample preparation methods for HS-SPME GC–MS analysis containing cut, pulping, milling and so on. All these methods may cause the loss of volatile compounds. In this study, cryogenic grinding was employed and the particle size (20, 40, 60, 80 mesh) was optimized. As shown in Fig. 1d, when the particle size was smaller than 40 mesh, the total content of volatile compounds was decreased significantly. This result indicated that the volatile compounds are easily lost in sample preparation procedure. The samples with particle size bigger than 20 mesh was not investigated in this study, because the large particle size might affect sampling uniformity. The total content of volatile compounds detected at 20 and 40 mesh was similar, but the particle size at 20 mesh requires less sample preparation time and lower cost. Therefore, 20 mesh was selected as optimum particle size.

### Volatile compounds in different parts of *A. villosum*

**Fig. 2:**
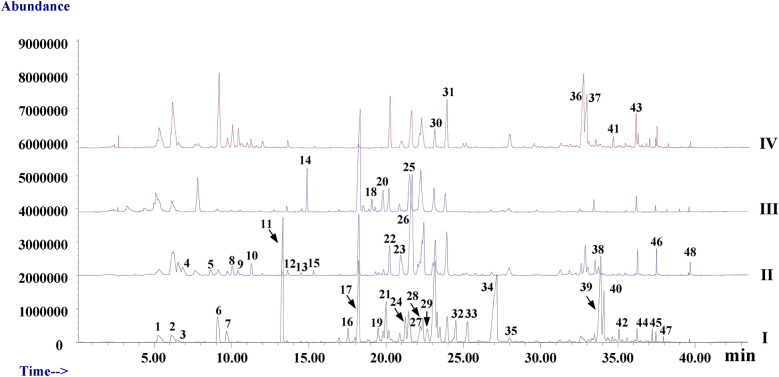
The chromatogram of representative *A. villosum* samples. I, fruit (sample 1); II, root (sample 19); III, leaf (sample 37); IV, stem (sample 55)

The volatile compounds in different parts of *A. villosum* were analyzed by the developed cryogenic grinding combined HS–SPME–GC–MS method. Volatile compounds were identified based on the National Institute of Standards and Technology (NIST) 2.0 Mass Spectra Database and the published literatures [1–8, 11–14]. The identified compounds were listed in Table 2 and Additional file 1: Table S1, and chromatograms of representative samples were shown in Fig. 2. (The chromatograms of all analyzed samples were shown in Additional file 1: Fig. S1.). As shown in Table 2, a total 48 main compounds were identified in different parts of *A. villosum* samples, accounting for 81.28–98.80% of the total detected essential oil components. Specifically, 33 compounds accounting for average 95.35% (range 90.86–98.80%) of the total detected essential oil components in fruit samples were identified, 27 compounds (93.60%, 89.79–98.67%) were identified in root samples, 19 compounds (86.00%, 81.28–93.78%) were identified in leaf samples and 21 compounds (92.72%, 90.33–98.51%) were identified in stem samples.

**Table 2:** The main volatile compounds in different parts of *A. villosum* samples

Codes	RT (min)	Compounds	Fruits		Roots		Leaves		Stems	
			Comparative index^a^	Relative content (%)^b^	Comparative index	Relative content (%)	Comparative index	Relative content (%)	Comparative index	Relative content (%)
1	5.26	(-)-β-Pinene	18.64^c^ (8.96–29.96)^d^	1.49^e^ (0.72–2.79)^f^	6.75 (2.61–13.10)	0.87 (0.33–1.55)	51.18 (19.99–100.00)	7.41 (2.66–17.96)	17.52 (5.39–42.82)	3.00 (1.88–4.71)
2	6.12	d-Limonene	21.41 (11.16–35.39)	1.49 (0.71–2.87)	18.60 (7.31–33.11)	2.05 (0.81–4.67)	47.97 (21.88–73.28)	5.87 (2.64–8.60)	53.19 (24.83–100.00)	8.62 (4.82–13.12)
3	6.48	Trans-β-ocimene	61.39 (24.13–100.00)	0.38 (0.16–0.69)	–	–	–	–	–	–
4	6.79	γ	–	–	66.32 (46.84–100.00)	1.67 (0.88–2.76)	–	–	–	–
5	8.65	Terpinolene	–	–	53.22 (21.67–100.00)	0.79 (0.23–1.58)	–	–	–	–
6	9.10	Camphor	56.8 (29.13–100.00)	3.91 (2.44–6.32)	6.68 (2.65–14.75)	0.77 (0.21–1.74)	–	–	48.92 (18.29–89.18)	8.08 (4.97–11.59)
7	9.69	Borneol	62.87 (34.65–100.00)	1.06 (0.54–1.72)	–	–	–	–	–	–
8	9.99	4-Terpineol	–	–	29.24 (17.60–46.87)	1.13 (0.67–90)	–	–	64.35 (34.96–100.00)	3.99 (1.08–6.49)
9	10.37	Terpineol	–	–	22.47 (13.15–44.24)	0.73 (0.39–1.61)	–	–	61.21 (34.34–100.00)	3.07 (1.74–5.23)
10	11.23	Fenchyl acetate	–	–	60.23 (36.83–100.00)	0.75 (0.55–1.24)	–	–	43.2 (14.71–74.93)	0.82 (0.49–1.20)
11	13.30	Bornyl acetate	70.76 (44.10–100.00)	10.53(8.14–14.27)	2.79 (0.77–8.38)	0.66 (0.21–2.31)	–	–	–	–
12	13.57	Methyl 2,5-octadecadiynoate	7.14 (2.96–16.60)	0.15 (0.07–0.40)	26.05 (8.13–48.86)	0.93 (0.24–1.77)	54.9 (24.02–100.00)	2.19 (0.93–5.55)	19.13 (5.14–41.33)	1.13 (0.24–2.90)
13	14.45	Myrtenyl acetate	–	–	25.02 (14.68–47.02)	0.26 (0.14–0.48)	55.52 (21.41–100.00)	0.65 (0.29–1.30)	–	–
14	14.86	Elixene	–	–	–	–	50.26 (26.48–100.00)	4.20 (2.48–6.05)	–	–
15	15.30	δ-Elemene	–	–	51.81 (25.62–100.00)	0.42 (0.24–0.71)	–	–	–	–
16	17.55	Naphthalene, 1,2,3,4,4a,7-Hexahydro-1,6-dimethyl-4-(1-methylethyl)-	72.73 (48.94–100.00)	0.94 (0.73–1.05)	–	–	–	–	–	–
17	18.22	Caryophyllene	38.73 (25.26–55.42)	8.70 (6.64–10.99)	24.15 (15.12–35.60)	8.67 (5.53–11.27)	70.29 (40.21–100.00)	27.67 (21.49–35.15)	1.85 (0.74–4.15)	1.00 (0.45–1.94)
18	19.08	Aromadendrene	–	–	–	–	52.71 (33.94–100.00)	1.62 (1.06–2.28)	–	–
19	19.49	Epi-β-Santalene	64.45 (38.13–100.00)	1.23 (0.86–1.72)	–	–	15.75 (9.48–26.90)	0.52 (0.37–0.74)	–	–
20	19.79	α-Caryophyllene	25.50 (16.51–38.79)	0.78 (0.60–1.27)	–	–	74.81 (41.95–100.00)	4.03 (3.06–5.88)	–	–
21	20.01	(Z)-β-Farnesene	80.18 (57.95–100.00)	3.53 (3.01–3.92)	–	–			–	–
22	20.18	Allo-aromadendrene	17.07 (7.12–36.64)	0.94 (0.35–2.23)	48.74 (28.44–69.09)	4.21 (2.86–5.87)	37.53 (15.95–69.46)	3.46 (2.23–4.68)	61.25 (25.84–100.00)	8.09 (5.70–10.83)
23	20.89	Unknown	23.66 (10.14–44.43)	0.94 (0.37–1.61)	59.46 (35.67–100.00)	3.74 (2.67–4.96)	21.18 (13.98–45.33)	1.48 (1.03–2.15)	19.89 (3.67–54.19)	1.79 (0.58–6.67)
24	21.26	γ-Muurolene	73.6 (48.74–100.00)	2.00 (1.48–2.27)	–	–	–	–	–	
25	21.46	α-Curcumene	25.37 (18.83–34.15)	2.30 (1.70–3.04)	–	–	61.83 (33.69–100.00)	9.76 (6.52–12.77)	41.76 (15.22–83.16)	8.73 (5.11–11.81)
26	21.59	Chamigrene	–	–	55.5 (37.63–100.00)	27.88 (22.26–36.38)	–	–	–	–
27	22.22	α-Amorphene	69.06 (41.43–100.00)	1.98 (1.07–2.91)	–	–	–	–	–	–
28	22.27	β-Guaiene	22.05 (10.59–35.77)	2.29 (0.99–3.47)	59.75 (37.91–100.00)	9.85 (7.27–13.71)	–	–	28.87 (3.58–61.43)	6.79 (1.20–9.99)
29	22.65	Cis-α-bisabolene	78.01 (53.60–100.00)	1.31 (0.91–1.87)	–	–	–	–	–	–
30	23.14	β-Bisabolene	77.31 (54.49–100.00)	11.50 (9.00–14.05)	26.6 (17.82–50.60)	6.26 (3.97–10.97)	14.38 (6.28–28.89)	3.62 (2.06–5.04)	10.34 (0.69–53.3)	3.09 (0.45–10.16)
31	23.93	δ-Cadinene	39.71 (27.76–54.46)	3.02 (1.93–3.89)	49.43 (28.41–73.14)	5.94 (3.97–8.28)	40.7 (24.71–74.94)	5.47 (3.00–13.52)	47.06 (6.08–100.00)	8.07 (1.49–10.29)
32	24.51	α-Patchoulene	70.09 (47.42–100.00)	2.10 (1.59–2.92)	–	–	–	–	–	–
33	25.26	Germacrene B	72.06 (48.84–100.00)	1.84 (1.46–2.34)	–	–	–	–	–	–
34	27.08	(E)-nerolidol	63.26 (36.22–100.00)	14.82 (10.14–18.66)	–	–	–	–	–	–
35	27.97	Caryophyllene oxide	18.99 (10.86–36.30)	0.59 (0.34–1.24)	61.34 (40.3–100.00)	3.01 (1.93–4.33)	21.34 (8.18–30.64)	1.15 (0.59–1.72)	35.87 (14.38–85.84)	2.71 (0.96–8.45)
36	32.57	γ-Eudesmol	–	–	12.93 (5.27–32.45)	2.17 (0.90–5.49)	–	–	52.87 (28.04–100.00)	12.84 (9.42–15.4)
37	32.86	Eremophilene	–	–	49.37 (32.64–78.66)	3.98 (2.46–6.07)	–	–	51.63 (22.49–100.00)	5.94 (4.25–8.64)
38	33.48	Longifolenaldehyde	–	–	35.29 (20.21–58.56)	1.55 (0.66–2.89)	62.82 (26.75–100.00)	2.91 (1.28–6.28)	–	–
39	33.90	Cubenol	69.36 (44.6–100.00)	10.04 (7.19–12.99)	6.83 (2.80–15.19)	1.54 (0.84–2.52)	–	–	–	–
40	34.09	Santalol,cis,α-	74.43 (42.5–100.00)	3.22 (2.13–4.11)	–	–	–	–	–	–
41	34.63	Widdrol	–	–	–	–	–	–	48.86 (15.39–100.00)	0.88 (0.23–2.17)
42	35.07	Acetic acid, 3-hydroxy-6-isopropenyl-4,8a-dimethyl-1,2,3,5,6,7,8,8a-octahydronaphthalen-2-yl ester	68.71 (39.22–100.00)	0.58 (0.39–0.79)	–	–	–	–	–	–
43	36.08	4-(2-Acetyl-5,5-dimethylcyclopent-2-enylidene)butan-2-one	–	–	–	–	–	–	57.82 (18.21–100.00)	2.69 (0.63–3.92)
44	36.23	Aristolene epoxide	22.27 (11.54–37.85)	0.65 (0.30–1.19)	27.11 (11.47–52.64)	1.32 (0.42–2.86)	58.48 (27.00–100.00)	3.02 (1.54–6.58)	–	–
45	37.20	Nerolidyl acetate	68.47 (37.48–100.00)	0.42 (0.25–0.55)	–	–	–	–	–	–
46	37.46	6-(1-Hydroxymethylvinyl)-4,8a-dimethyl-3,5,6,7,8,8a-hexahydro-1H-naphthalen-2-one	20.25 (11.91–46.81)	0.37 (0.22–0.94)	67.34 (39.72–100.00)	2.00 (1.13–3.37)	20.22 (13.75–30.53)	0.66 (0.42–1.31)	26.64 (9.35–49.58)	1.23 (0.33–2.84)
47	37.95	Santalol	59.35 (34.97–100.00)	0.13 (0.08–0.22)	–	–	–	–	–	–
48	39.50	Cembrene	19.97 (8.67–34.40)	0.09 (0.05–0.16)	65.38 (44.24–100.00)	0.46 (0.30–0.82)	38.9 (19.09–58.49)	0.31 (0.14–0.52)	15.37 (5.73–35.40)	0.16 (0.03–.31)
Total			77.81 (55.84–100.00)	95.35 (90.86–98.80)	47.95 (35.68–68.76)	93.60 (89.79–98.67)	39.88 (29.49–56.66)	86.00 (81.28–93.78)	32.32 (16.64–57.90)	92.72 (90.33–98.51)
		Chemical classes								
		Monoterpene hydrocarbons	26.87 (14.60–41.69)	3.37 (1.81–6.09)	26.75 (18.39–38.77)	5.38 (3.38–8.63)	59.29 (25.78–100.00)	13.29 (5.60–23.9)	40.70 (17.68–79.34)	11.62 (7.80–16.32)
		Oxygenated monoterpenes	68.75 (42.38–100.00)	15.51 (11.77–19.95)	11.72 (7.00–20.14)	4.30 (2.83–8.45)	1.58 (0.61–2.84)	0.65 (0.29–1.30)	28.69 (14.99–45.09)	15.97 (11.74–22.37)
		Sesquiterpene hydrocarbons	70.02 (48.17–95.35)	44.89 (34.4–50.98)	66.34 (48.55–100.00)	67.21 (61.31–73.02)	54.17 (34.12–83.97)	60.34 (52.85–68.94)	28.52 (11.79–59.51)	41.7 (25.26–49.12)
		Oxygenated sesquiterpenes	67.57 (40.51–100.00)	29.81 (21.3–36.02)	16.17 (9.56–20.54)	11.58 (8.22–15.2)	9.91 (5.35–15.28)	7.75 (5.19–14.76)	16.86 (9.09–32.48)	17.66 (12.69–21.5)
		Others	37.19 (19.16–54.55)	1.76 (1.09–2.56)	67.99 (43.35–100.00)	5.13 (4.24–6.52)	46.48 (27.63–85.52)	3.97 (2.32–7.29)	50.38 (21.39–88.52)	5.77 (2.40–10.84)

^a^ The comparative index was calculated as: comparative index (A/SI) = P(A/SI)/P(A_Max_.)*100; (comparative index (A/SI) was the comparative index of compound A in sample SI; P(A/SI) was the peak area of compound A in sample SI; P(A_Max_.) was the maximum peak area of compound A in different parts of *A. villosum* samples)

^b^ Relative content was calculated as: relative content (A/SI) = P(A/SI)/P(SI)*100%; (relative content (A/SI) was the relative content of compound A in sample SI; P(A/SI) was the peak area of compound A in sample SI; P(SI) was the total peak area of detected volatile compounds in sample SI)

^c^ Average comparative index (n = 18)

^d^ Range of comparative index (n = 18)

^e^ Average relative content (n = 18)

^f^ Range of comparative index (n = 18)

All data was presented as average of two determinations, their relative average deviations were less than 5.3%

The volatile compounds from fruits of *A. villosum* were characterized by high amounts of sesquiterpenes hydrocarbons (44.89%, 34.4–50.98%) followed by oxygenated sesquiterpenes (29.81%, 21.3–36.02%), oxygenated monoterpenes (15.51%, 11.77–19.95%) and monoterpene hydrocarbons (3.37%, 1.81–6.09%). The most abundant components were camphor (3.91%, 2.44–6.32%), bornyl acetate (10.53%, 8.14–14.27%), caryophyllene (8.70%, 6.64–10.99%), β-bisabolene (11.50%, 9.00–14.05%), (E)-nerolidol (14.82%, 10.14–18.66%) and cubenol (10.04%, 7.19–12.99%).

In our previous work, the volatile compounds in 12 batches of the dried fruit of *A. villosum* were evaluated and 11 main components were determined. Camphor, borneol and bornyl acetate were the most abundant compounds, accounting for almost 98% of the determined compounds [1]. This result was in accordance with that previously reported by other authors. Zeng et al. (2010) analyzed the volatile compounds in different varieties of Amomi Fructus and found camphor (27.81%), borneol (2.11%) and bornyl acetate (59.60%) as the major components in dried fruit of *A. villosum* [31]. However, in fresh fruits of *A. villosum*, the average content of camphor, borneol and bornyl acetate was 3.91%, 1.06% and 10.53%, respectively. This result is quite different with that of dried fruits of *A. villosum.* The reason might be that many volatile components are lost or degraded during drying and storage. (E)-nerolidol, the most abundant component in fresh fruits of *A. villosum*, exhibits many biological functions such as antifungal [32] and antiulcer [33] effects. However, this compound was great lost in dried samples. Therefore, qualitative and comparative analysis of volatile compounds in fresh *A. villosum* samples can obtain much more information which is helpful for their performance in the fields of functional foods, agriculture and biomedical industry.

The volatile compounds from root samples showed a higher amount of sesquiterpene hydrocarbons (67.21%, 61.31–73.02%). Oxygenated sesquiterpenes (11.58%, 8.22–15.2%) represented the second most abundant chemical classes of this part. The most abundant components were caryophyllene (8.67%, 5.53–11.27%), allo-aromadendrene (4.21%, 2.86–5.87%), chamigrene (27.88%, 22.26–36.38%), β-guaiene (9.85%, 7.27–13.71%), β-bisabolene (6.26%, 3.97–10.97%), δ-cadinene (5.94%, 3.97–8.28%) and eremophilene (3.98%, 2.46–6.07%) as the major component. Monoterpene hydrocarbons (5.81%, 4.03–8.90%) and oxygenated monoterpenes (4.30%, 2.83–8.45%) gave a minor contribution.

The volatile compounds from leaf samples consisted mostly of sesquiterpene hydrocarbons (60.34%, 52.85–68.94%) and monoterpene hydrocarbons (13.29%, 5.60–23.9%) followed by oxygenated sesquiterpenes (7.75%, 5.19–14.76%). The most abundant components were (-)-β-pinene (7.41%, 2.66–17.96%), d-limonene (5.87%, 2.64–8.60%), elixene (4.20%, 2.48–6.05%), caryophyllene (27.67%, 21.49–35.15%), α-caryophyllene (4.03%, 3.06–5.88%), α-curcumene (9.76%, 6.52–12.77%), δ-cadinene (5.47%, 3.00–13.52%).

High content of sesquiterpene hydrocarbons (41.70%, 25.26–49.12%) were detected in stem samples, followed by oxygenated sesquiterpenes (17.66%, 12.69–21.50%), oxygenated monoterpenes (15.97%, 11.74–22.37%), monoterpene hydrocarbons (11.62%, 7.80–16.32%). d-limonene (8.62%, 4.82–13.12%), camphor (8.08%, 4.97–11.59%), 4-terpineol (3.99%, 1.08–6.49%), allo-aromadendrene (8.09%, 5.70–10.83%), α-curcumene (8.73%, 5.11–11.81%), β-guaiene (6.79%, 1.20–9.99%), δ-cadinene (8.07%, 1.49–10.29%), γ-eudesmol (12.84%, 9.42–15.4%) and eremophilene (5.94%, 4.25–8.64%) were the most abundant components.

**Fig. 3:**
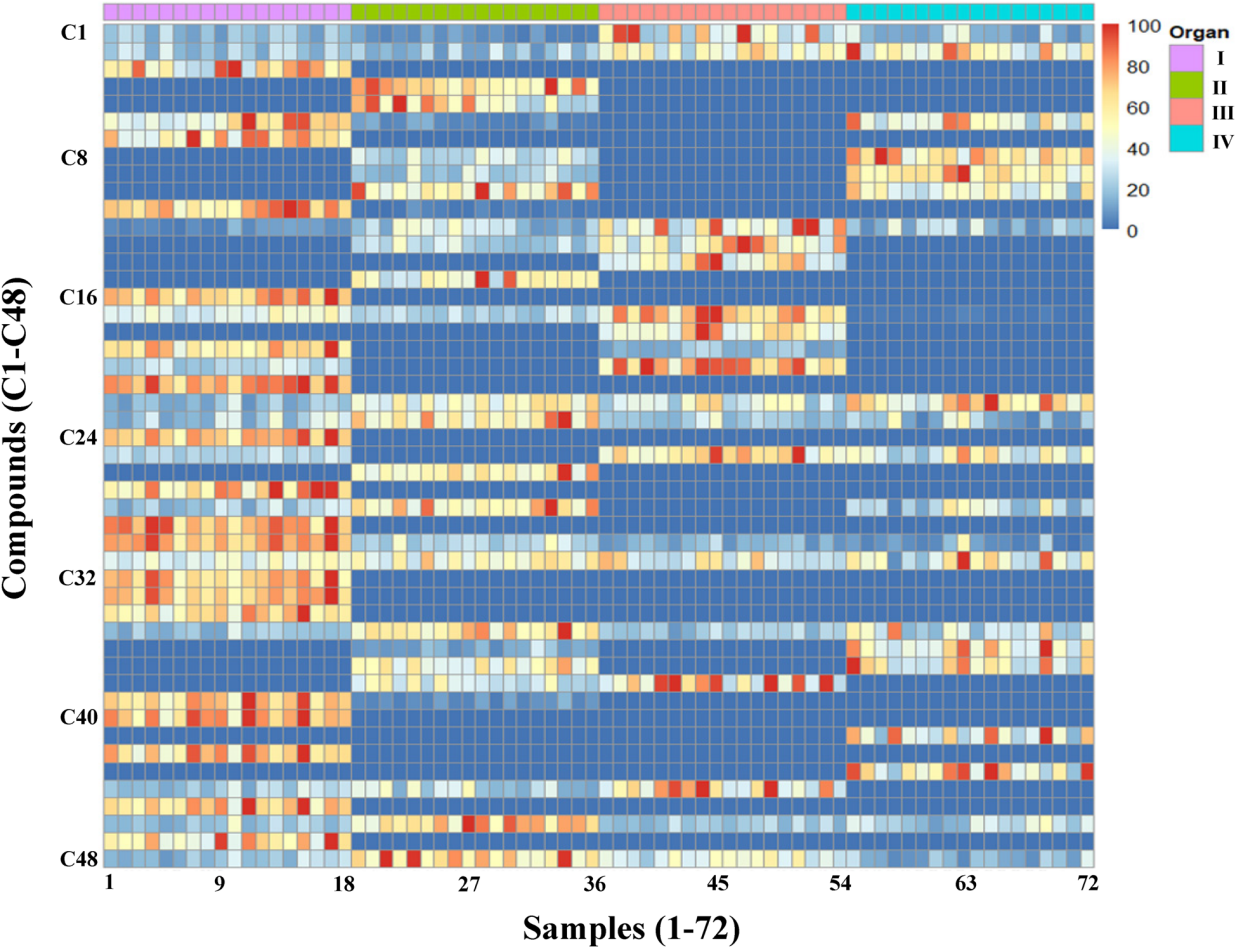
Heatmap analysis of 72 analyzed samples. I, fruit samples; II, root samples; III, leaf samples; IV, stem samples

**Fig. 4:**
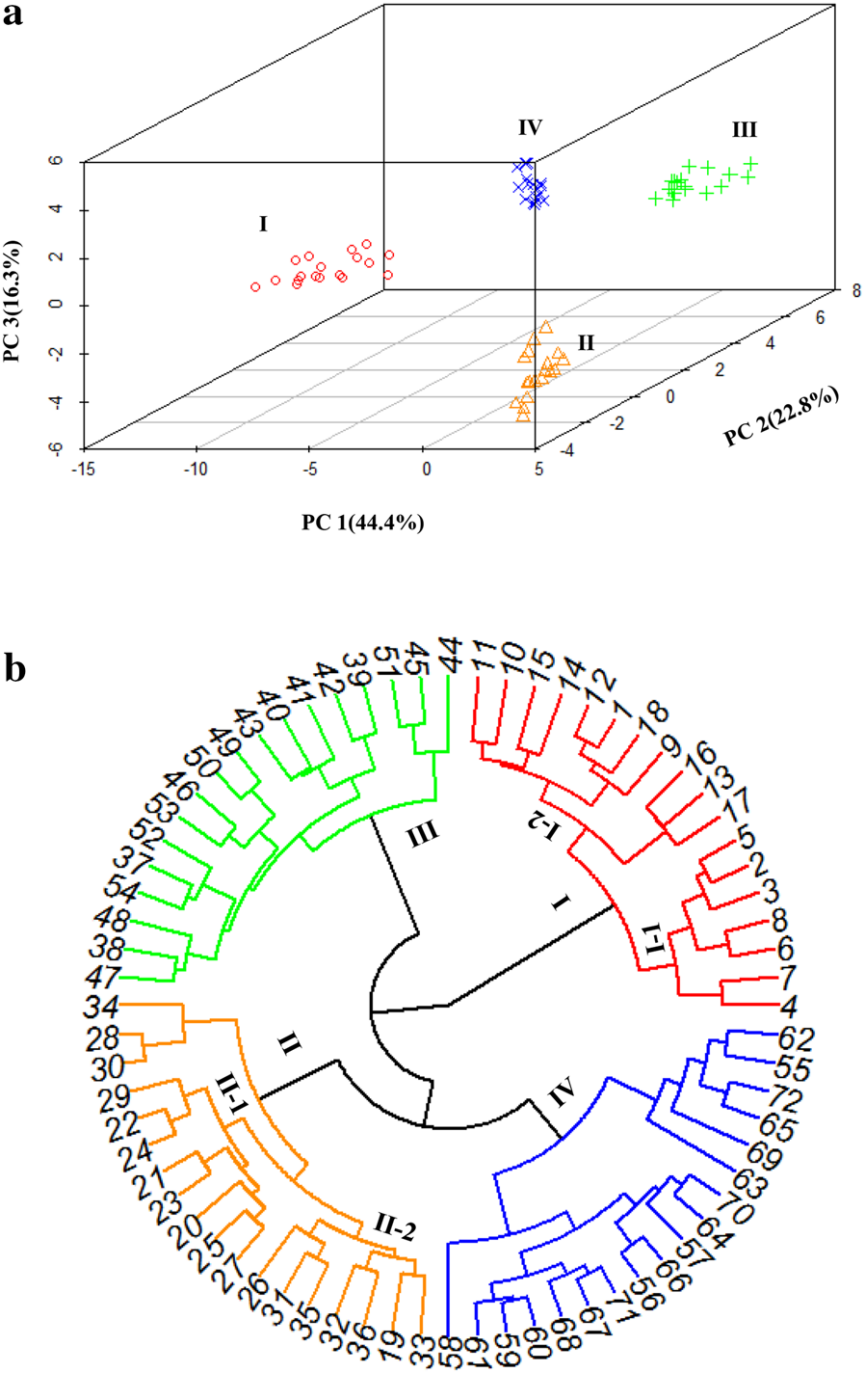
**a** Classification of 72 analyzed samples by PCA analysis. **b** Dendrograms of 72 analyzed samples resulting from hierarchical clustering analysis. I, fruit samples; II, root samples; III, leaf samples; IV, stem samples

For further evaluation, the comparative index was used for comparative analysis of volatile compounds in different parts of *A. villosum* samples. The comparative index was calculated as: comparative index (A/SI) = P(A/SI)/P(A_Max._)*100; (comparative index (A/SI) was the comparative index of compound A in sample SI; P(A/SI) was the peak area of compound A in sample SI; P(A_Max._) was the maximum peak area of compound A in different parts of *A. villosum* samples). As shown in Table 2, the fruits of *A. villosum* showed high amounts of volatile compounds (average 77.81, range 55.84–100) followed by roots (47.95, 35.68–68.76), leaves (39.88, 29.49–56.66) and stems (32.32, 16.64-57.90). Heatmap analysis was performed on 72 *A. villosum* samples with 48 identified volatile compounds and their comparative index (Fig. 3). As different parts of the same plant, there are many common components between fruit and other parts (Fig. 3). The bioactive compounds such as (-)-β-pinene, d-limonene and caryophyllene were existed in four parts of *A. villosum.* Additionally, the main pharmacological active ingredients camphor can be detected in root and stem samples, and bornyl acetate can be detected in root samples. These results indicated that other parts of *A. villosum* have potential for partial replacement of the fruit. In addition, there are also many characteristic components detected in root, leaf and stem of *A. villosum*. Higher content of chamigrene (55.5, 37.63–100.00) and β-guaiene (59.75, 37.91–100.00) were detected in root samples. Elixene (50.26, 26.48–100.00), α-caryophyllene (74.81, 41.95–10.00) and α-curcumene (61.83, 33.69–100.00) were mostly existed in leaf samples. The stem samples contain higher amounts of allo-aromadendrene (61.25, 25.84–100.00) and γ-eudesmol (52.87, 28.04–100.00). These results are beneficial for development of the unique applications of different parts of *A. villosum* in biomedical and functional foods industry. As sample distance assessment characterized by the volatile compounds profile of each sample is a useful way to depict the relationships between samples, PCA and HCA were performed to determine the similarity among samples derived from different parts of *A. villosum*. The PCA and HCA were performed on 72 *A. villosum* samples with 48 identified volatile compounds and their comparative index. As shown in Fig. 4a, three principal components (PC) explained 83.5% of the total variance. Specifically, PC 1, 2 and 3 were account for 44.4%, 22.8% and 16.3, respectively. All the *A. villosum* samples could be divided into four groups, which were corresponding to sample sets collected from fruit, root, leaf and stem, respectively. The HCA (Fig. 4b) was in accordance with that of PCA, four parts of *A. villosum* samples were divided into four subgroups clearly. These results indicated that the pattern of the content of volatile compounds detected in different organs of *A. villosum* were so divergent that a sample could easily be assigned to its sample set basing on this kind of pattern.

### Comparison of volatile compounds in fresh *A. villosum* from different geographical areas

**Fig. 5:**
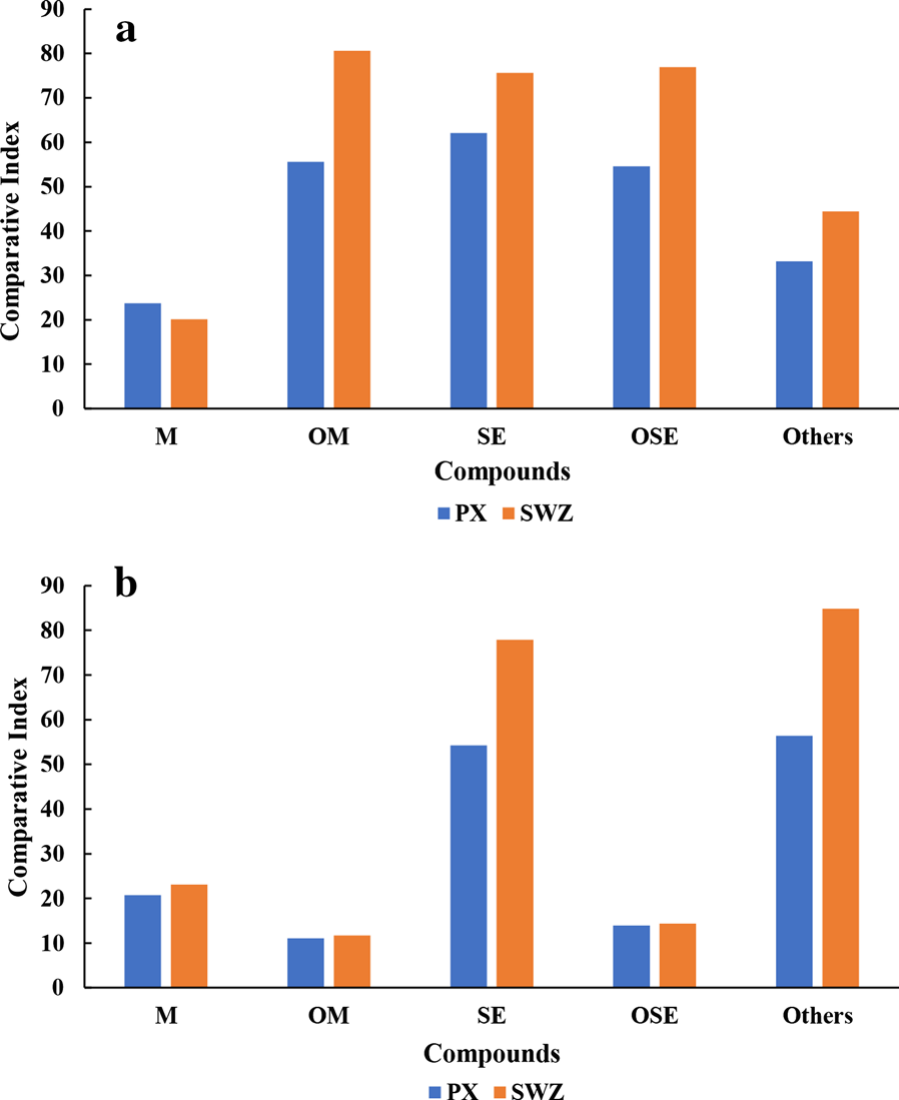
Comparative analysis of five chemical classes of *A. villosum* samples from different regions. **a** Fruit samples **b** root samples. M: monoterpene hydrocarbons; OM: oxygenated monoterpenes; SE: sesquiterpene hydrocarbons; OSE: oxygenated sesquiterpenes; PX: Pingxi village; SWZ, Shiwanzai village. All data was presented as average of two determinations, their relative average deviations were less than 5.3%

As shown by the results of HCA (Fig. 4b), samples collected from fruits could be divided into two groups although two of the samples (sample 1 and 9) was misassigned. Samples in group I-1 were all from Pingxi village, Yangchun and samples in group I-2 were from Shiwanzai village, Yangchun (exclude sample 1 and 9). This result indicated that the volatile compounds in fruit samples may be affected by the growing region. The average comparative index of five chemical classes (monoterpene hydrocarbons, oxygenated monoterpenes, sesquiterpene hydrocarbons, oxygenated sesquiterpenes and others) of group I–1 and I–2 were shown in Fig. 5a. The results showed that great difference was observed in four chemical classes (oxygenated monoterpenes, sesquiterpene hydrocarbons, oxygenated sesquiterpenes and others). This indicated that these four chemical classes in fruit of *A. villosum* might be more susceptible to the growing environment. Similarly, many root samples from different regions were also divided into two groups (group II-1 and group II-2). As shown in Fig. 5b great difference was observed in sesquiterpene hydrocarbons and other compounds. Leaf and stem samples could not be clustered into any subgroups that related to the geographic distribution of the corresponding samples. These results indicated that the fruits and roots of *A. villosum* produced by distinct areas, at least the areas analyzed here, showed a difference in the content of volatile compounds. However, for further investigate, much more samples are needed to investigate the most suitable growth environment, cultivating mode and collection time for *A. villosum* with high quality.

## Conclusions

In this study, the volatile compounds in different parts of fresh *A. villosum* from different origins were systemic analyzed and compared by using cryogenic grinding combined HS–SPME–GC–MS for the first time. The type and content of main volatile compounds in fresh fruits of *A. villosum* is quite different with that of dried samples (reported in previous studies). This indicated that systemic analysis of fresh *A. villosum* can obtain much more information which is helpful for their development and application. Other parts of *A. villosum* have potential for partial replacement of the fruit, due to many common components with biological activities were detected in fruits and other parts of *A. villosum*. In addition, many characteristic components were also detected in different parts of *A. villosum*. These results are beneficial for development of the unique applications of different parts of *A. villosum*. Additionally, fruit and root samples also could be divided into two subgroups (HCA) in accordance with their regions. Furthermore, our research is helpful for comprehensive utilization and quality control of *A. villosum*.

## Supplementary information

**Additional file 1: Fig.S1.** The chromatogram of *A. villosum* samples. I, fruit (S1-S18); II, root (S19-S36); III, leaf (S37-S54); IV, stem (S55-S72). **Table S1.** The main volatile compounds in 72 *A. villosum* samples.

## Data Availability

The research data generated from this study is included within the article.

## Abbreviations

CAR/PDMS: Carboxen poly(dimethylsiloxane)
EI: Electron-impact mode
HS-SPME: Headspace solid-phase micro-extraction
HCA: Hierarchical clustering analysis
RPA: Relative peak area
GC–MS: Gas chromatography–mass spectrometry
PCA: Principle components analysis
PDMS: Polydimethylsiloxane
PDMS/DVB: Polydimethylsiloxane/divinylbenzene
