# Supplementary material for: Comparison of volatile compounds in different parts of fresh Amomum villosum Lour. from different geographical areas using cryogenic grinding combined HS–SPME–GC–MS
Source: Chin Med. 2020 Sep 7;15:97. doi: 10.1186/s13020-020-00377-z (PMC7487758; doi:10.1186/s13020-020-00377-z)
Supplement: Supplementary file 1 — Additional file 1: Fig.S1. The chromatogram of A. villosum samples. I, fruit (S1-S18); II, root (S19-S36); III, leaf (S37-S54); IV, stem (S55-S72). Table S1. The main volatile compounds in 72 A. villosum samples. [file 13020_2020_377_MOESM1_ESM.docx]

**Supplementary Material**

**Comparison of volatile compounds** **in different parts of** **fresh *Amomum villosum* Lour. from different** **geographical areas using** **cryogenic grinding combined HS-SPME-GC-MS**

**Ling-Xiao Chen,^a1^ Yun-Feng Lai,^a1^ Wei-Xiong Zhang,^a,b^ Jing Cai,^b^ Hao Hu,^a^* Ying Wang,^a^ Jing Zhao,^a^* Shao-Ping Li^a^ ***

^a^ State Key Laboratory of Quality Research in Chinese Medicine, Institute of Chinese Medical Sciences, University of Macau, Macao SAR, China

^b^ Center for Ecological and Environmental Science, Northwestern Polytechnical University, Xi^’^an 710072, China

^1^The authors contributed the works equally.

* Correspondence: E-mail: Haohu@umac.edu.mo (H. Hu);

jingzhao@umac.edu.mo/zhaojing.cpu@163.com (J. Zhao);

spli@umac.edu.mo/Lishaoping@hotmail.com; (S. P. Li); Tel.: +853 88224692.


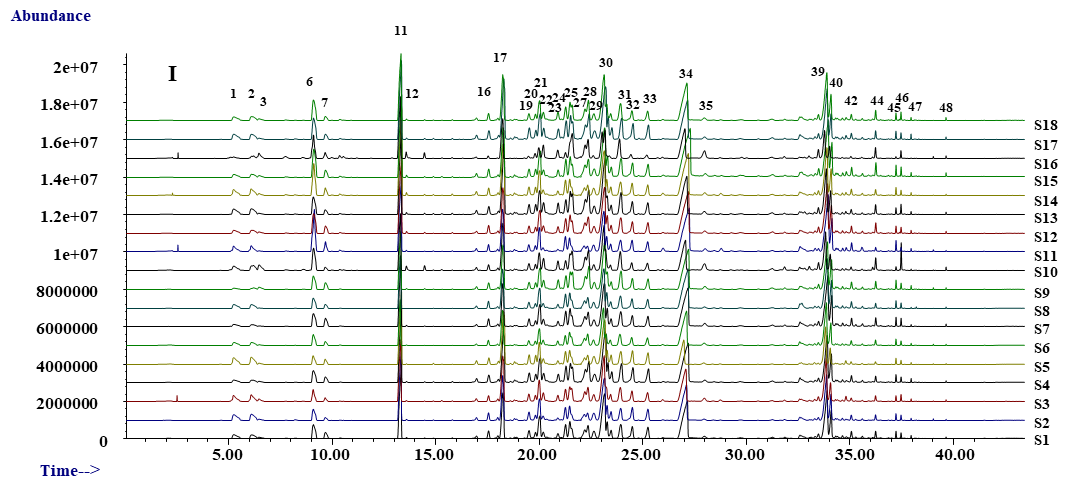


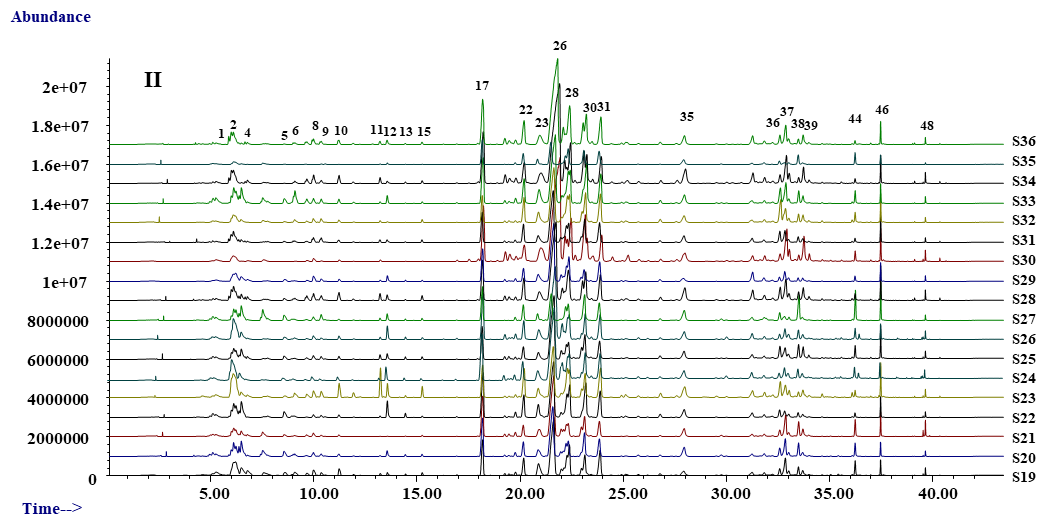


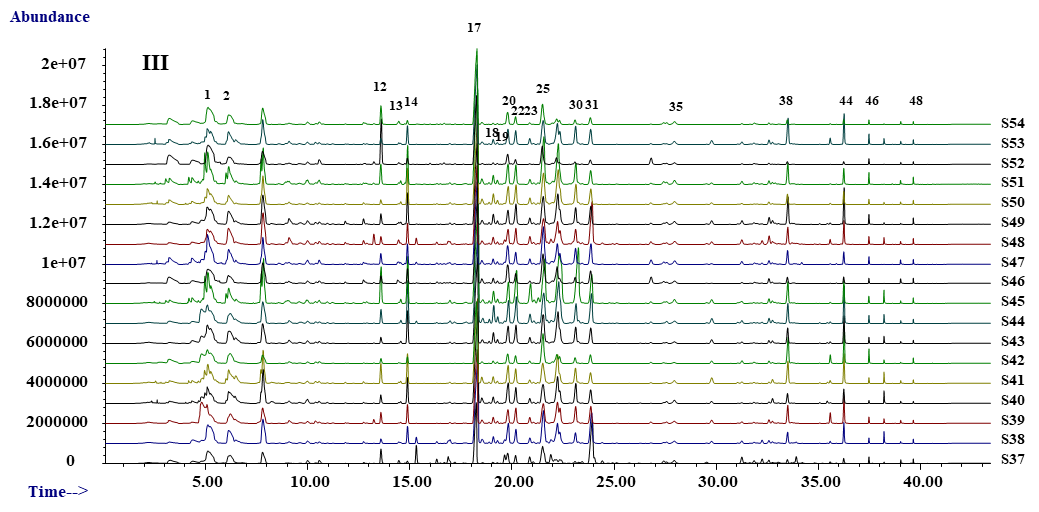


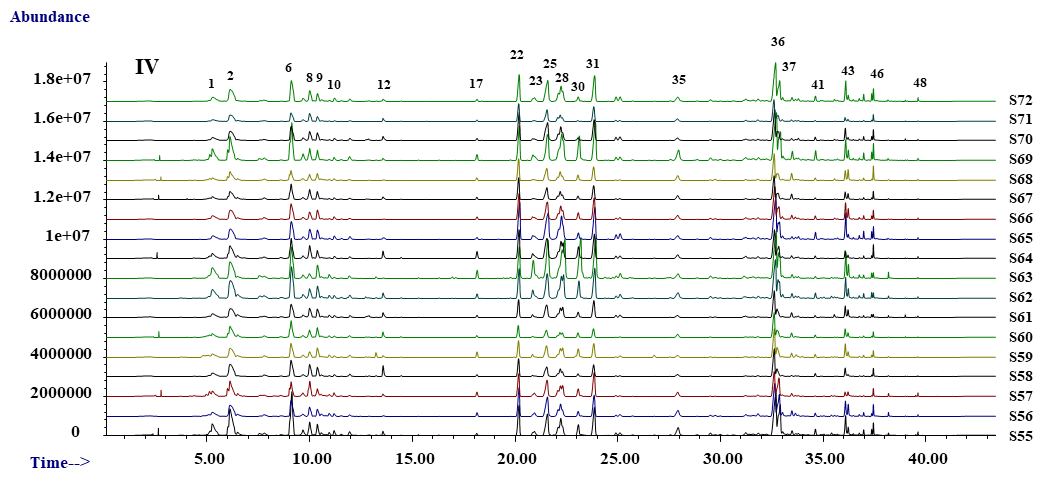


Fig.S1 The chromatogram of *A. villosum* samples. I, fruit (S1-S18); II, root (S19-S36); III, leaf (S37-S54); IV, stem (S55-S72).

**Table S1. The main volatile compounds in 72 *Amomum villosum* samples.**

| Code | RT（min） | Compounds | Sample | | | | | | | | | | | | | | | | | | |
| --- | --- | --- | --- | --- | --- | --- | --- | --- | --- | --- | --- | --- | --- | --- | --- | --- | --- | --- | --- | --- | --- |
|  |  |  | 1 | | 2 | 3 | 4 | 5 | 6 | 7 | 8 | 9 | 10 | 11 | 12 | 13 | 14 | 15 | 16 | 17 | 18 |
| 1 | 5.26 | (-)-β-pinene | 18.24^a^ (1.50)^b^ | | 27.63 (2.42) | 23.01 (2.12) | 11.32 (0.82) | 29.96 (2.79) | 13.83 (1.52) | 10.19 (0.81) | 19.09 (1.69) | 8.96 (0.72) | 22.58 (1.97) | 18.41 (1.30) | 14.61 (1.07) | 25.91 (1.79) | 24.81 (1.70) | 14.84 (0.92) | 9.67 (0.77) | 25.23 (1.59) | 17.25 (1.39) |
| 2 | 6.117 | D-limonene | 19.11 (1.36) | | 32.58 (2.48) | 28.82 (2.30) | 12.60 (0.79) | 35.39 (2.87) | 15.22 (1.45) | 12.16 (0.84) | 19.81 (1.53) | 11.68 (0.81) | 28.39 (2.16) | 20.71 (1.27) | 11.16 (0.71) | 30.66 (1.84) | 29.13 (1.74) | 17.01 (0.92) | 12.54 (0.87) | 28.72 (1.57) | 19.68 (1.38) |
| 3 | 6.483 | trans-β-ocimene | 57.50 (0.37) | | 60.26 (0.42) | 89.04 (0.65) | 43.82 (0.25) | 58.05 (0.43) | 31.11 (0.27) | 26.83 (0.17) | 24.13 (0.17) | 93.10 (0.59) | 100.00 (0.69) | 29.21 (0.16) | 71.04 (0.41) | 56.62 (0.31) | 78.95 (0.43) | 88.42 (0.43) | 78.95 (0.50) | 51.33 (0.25) | 66.58 (0.42) |
| 4 | 6.786 | γ-terpinene | - | | - | - | - | - | - | - | - | - | - | - | - | - | - | - | - | - | - |
| 5 | 8.651 | terpinolene | - | | - | - | - | - | - | - | - | - | - | - | - | - | - | - | - | - | - |
| 6 | 9.095 | camphor | 45.73 (3.37) | | 35.54 (2.79) | 30.66 (2.53) | 40.28 (2.61) | 29.13 (2.44) | 32.66 (3.23) | 43.64 (3.10) | 33.51 (2.67) | 43.62 (3.14) | 68.77 (5.40) | 100.00 (6.32) | 65.80 (4.35) | 58.21 (3.61) | 92.07 (5.68) | 93.65 (5.23) | 68.58 (4.93) | 71.68 (4.05) | 68.90 (5.00) |
| 7 | 9.69 | borneol | 75.48 (1.34) | | 35.03 (0.67) | 36.72 (0.73) | 34.65 (0.54) | 57.48 (1.16) | 44.34 (1.06) | 100.00 (1.72) | 50.27 (0.97) | 82.23 (1.43) | 35.46 (0.67) | 89.38 (1.37) | 88.96 (1.42) | 56.92 (0.85) | 78.04 (1.16) | 85.86 (1.16) | 66.99 (1.16) | 65.37 (0.89) | 48.50 (0.85) |
| 8 | 9.998 | 4-terpineol | - | | - | - | - | - | - | - | - | - | - | - | - | - | - | - | - | - | - |
| 9 | 10.369 | terpineol | - | | - | - | - | - | - | - | - | - | - | - | - | - | - | - | - | - | - |
| 10 | 11.232 | fenchyl acetate | - | | - | - | - | - | - | - | - | - | - | - | - | - | - | - | - | - | - |
| 11 | 13.296 | bornyl acetate | 71.69 (11.22) | | 68.51 (11.44) | 60.25 (10.57) | 73.85 (10.16) | 80.16 (14.27) | 44.1 (9.26) | 61.18 (9.23) | 48.14 (8.14) | 55.32 (8.46) | 50.09 (8.35) | 77.55 (10.42) | 84.51 (11.86) | 90.46 (11.93) | 100.00 (13.11) | 92.69 (10.99) | 63.41 (9.68) | 85.33 (10.25) | 66.37 (10.24) |
| 12 | 13.57 | methyl 2,5-octadecadiynoate | 5.59 (0.13) | | 3.48 (0.08) | 5.23 (0.13) | 5.77 (0.12) | 3.67 (0.09) | 2.96 (0.09) | 6.05 (0.13) | 3.05 (0.07) | 3.32 (0.07) | 16.60 (0.40) | 5.56 (0.11) | 10.41 (0.21) | 10.56 (0.20) | 11.51 (0.22) | 10.43 (0.18) | 10.63 (0.24) | 6.03 (0.11) | 7.65 (0.17) |
| 13 | 14.45 | myrtenyl acetate | - | | - | - | - | - | - | - | - | - | - | - | - | - | - | - | - | - | - |
| 14 | 14.862 | elixene | - | | - | - | - | - | - | - | - | - | - | - | - | - | - | - | - | - | - |
| 15 | 15.302 | δ-elemene | - | | - | - | - | - | - | - | - | - | - | - | - | - | - | - | - | - | - |
| 16 | 17.548 | naphthalene, 1,2,3,4,4a,7-hexahydro-1,6-dimethyl-4-(1-methylethyl)- | 76.71 (1.04) | | 72.99 (1.05) | 55.79 (0.85) | 82.99 (0.99) | 67.43 (1.04) | 48.94 (0.89) | 77.92 (1.02) | 68.13 (1.00) | 68.42 (0.90) | 56.38 (0.81) | 63.23 (0.73) | 81.46 (0.99) | 89.35 (1.02) | 74.16 (0.84) | 89.44 (0.92) | 64.94 (0.86) | 100.00 (1.04) | 70.87 (0.94) |
| 17 | 18.222 | caryophyllene | 35.39 (8.33) | | 32.85 (8.25) | 36.77 (9.70) | 45.94 (9.50) | 37.08 (9.92) | 25.26 (7.97) | 32.44 (7.36) | 28.27 (7.19) | 37.21 (8.56) | 38.27 (9.60) | 32.87 (6.64) | 36.12 (7.62) | 55.42 (10.99) | 44.14 (8.70) | 43.55 (7.76) | 43.16 (9.91) | 55.41 (10.00) | 37.05 (8.59) |
| 18 | 19.08 | aromadendrene | - | | - | - | - | - | - | - | - | - | - | - | - | - | - | - | - | - | - |
| 19 | 19.491 | Epi-β-Santalene | 63.74 (1.27) | | 66.92 (1.43) | 56.28 (1.26) | 82.02 (1.44) | 75.62 (1.72) | 38.13 (1.02) | 61.56 (1.19) | 48.51 (1.05) | 61.41 (1.20) | 56.27 (1.20) | 50.35 (0.86) | 60.98 (1.09) | 82.74 (1.39) | 67.12 (1.12) | 69.48 (1.05) | 64.98 (1.27) | 100.00 (1.53) | 54.05 (1.06) |
| 20 | 19.794 | α-caryophyllene | 21.65 (0.69) | | 19.57 (0.67) | 23.17 (0.83) | 31.60 (0.89) | 34.90 (1.27) | 16.51 (0.71) | 19.70 (0.61) | 17.39 (0.60) | 25.07 (0.79) | 25.82 (0.88) | 22.18 (0.61) | 23.15 (0.67) | 38.79 (1.05) | 33.64 (0.90) | 29.03 (0.71) | 21.24 (0.66) | 32.28 (0.79) | 23.36 (0.74) |
| 21 | 20.006 | (Z)-β-farnesene | 83.05 (3.82) | | 79.17 (3.89) | 69.14 (3.57) | 96.82 (3.92) | 71.89 (3.76) | 57.95 (3.58) | 82.47 (3.66) | 72.97 (3.63) | 80.39 (3.62) | 61.37 (3.01) | 84.87 (3.35) | 88.19 (3.64) | 85.83 (3.33) | 92.38 (3.56) | 100.00 (3.49) | 68.53 (3.08) | 97.59 (3.45) | 70.61 (3.20) |
| 22 | 20.183 | allo-aromadendrene | 13.31 (0.76) | | 8.85 (0.54) | 19.43 (1.25) | 19.46 (0.98) | 11.14 (0.72) | 11.92 (0.91) | 16.03 (0.88) | 10.10 (0.62) | 20.11 (1.12) | 36.64 (2.23) | 7.12 (0.35) | 16.76 (0.86) | 25.74 (1.24) | 10.15 (0.49) | 14.73 (0.64) | 24.37 (1.36) | 23.63 (1.04) | 17.70 (1.00) |
| 23 | 20.892 | unknown | 14.82 (0.62) | | 10.68 (0.48) | 19.66 (0.93) | 32.51 (1.20) | 10.95 (0.52) | 14.22 (0.80) | 15.82 (0.64) | 12.76 (0.58) | 36.46 (1.50) | 35.86 (1.61) | 10.14 (0.37) | 20.94 (0.79) | 44.43 (1.57) | 12.17 (0.43) | 22.98 (0.73) | 36.79 (1.51) | 42.76 (1.38) | 32.00 (1.32) |
| 24 | 21.257 | γ-muurolene | 72.91 (2.08) | | 68.92 (2.10) | 63.04 (2.02) | 84.24 (2.11) | 68.26 (2.21) | 50.96 (1.95) | 81.61 (2.24) | 73.65 (2.27) | 70.75 (1.97) | 48.74 (1.48) | 80.62 (1.97) | 77.53 (1.98) | 74.17 (1.78) | 81.28 (1.94) | 95.62 (2.07) | 63.24 (1.76) | 100.00 (2.19) | 69.19 (1.95) |
| 25 | 21.457 | α-curcumene | 24.29 (2.29) | | 30.19 (3.04) | 18.83 (2.00) | 26.45 (2.20) | 26.03 (2.80) | 19.27 (2.44) | 25.67 (2.34) | 21.73 (2.22) | 24.38 (2.25) | 22.64 (2.28) | 23.58 (1.91) | 26.05 (2.21) | 34.15 (2.72) | 21.46 (1.70) | 28.82 (2.06) | 22.34 (2.06) | 34.13 (2.47) | 26.67 (2.48) |
| 26 | 21.594 | chamigrene | - | | - | - | - | - | - | - | - | - | - | - | - | - | - | - | - | - | - |
| 27 | 22.217 | α-amorphene | 55.21 (1.66) | | 45.02 (1.44) | 58.70 (1.98) | 86.85 (2.30) | 47.09 (1.61) | 46.06 (1.86) | 68.53 (1.99) | 51.21 (1.66) | 86.73 (2.55) | 81.08 (2.60) | 41.43 (1.07) | 70.31 (1.90) | 100.00 (2.53) | 52.83 (1.33) | 88.61 (2.02) | 99.34 (2.91) | 98.95 (2.28) | 65.11 (1.93) |
| 28 | 22.272 | β-guaiene | 20.44 (2.23) | | 10.77 (1.25) | 19.00 (2.32) | 26.28 (2.52) | 12.75 (1.58) | 14.61 (2.14) | 18.51 (1.94) | 15.86 (1.87) | 32.58 (3.47) | 26.04 (3.02) | 10.59 (0.99) | 24.50 (2.39) | 35.77 (3.28) | 13.19 (1.20) | 26.57 (2.19) | 24.46 (2.60) | 33.85 (2.83) | 31.16 (3.35) |
| 29 | 22.658 | cis-α-bisabolene | 85.57 (1.50) | | 89.62 (1.67) | 71.34 (1.40) | 98.97 (1.52) | 94.13 (1.87) | 55.20 (1.29) | 76.90 (1.30) | 66.03 (1.25) | 77.27 (1.32) | 56.91 (1.06) | 73.72 (1.11) | 78.91 (1.24) | 91.76 (1.35) | 81.78 (1.20) | 83.38 (1.10) | 53.60 (0.91) | 100.00 (1.34) | 69.02 (1.19) |
| 30 | 23.143 | β-bisabolene | 79.49 (12.29) | | 77.95 (12.86) | 81.02 (14.05) | 96.12 (13.06) | 79.17 (13.92) | 54.49 (11.3) | 73.66 (10.98) | 65.42 (10.92) | 78.42 (11.85) | 62.22 (10.25) | 67.80 (9.00) | 78.33 (10.86) | 92.57 (12.06) | 74.80 (9.68) | 84.24 (9.87) | 75.07 (11.32) | 100.00 (11.86) | 70.90 (10.80) |
| 31 | 23.931 | δ-cadinene | 35.35 (2.81) | | 29.36 (2.49) | 34.57 (3.08) | 46.86 (3.27) | 32.10 (2.90) | 27.76 (2.96) | 37.78 (2.90) | 30.66 (2.63) | 45.70 (3.55) | 45.88 (3.89) | 28.28 (1.93) | 39.85 (2.84) | 54.46 (3.65) | 34.27 (2.28) | 45.51 (2.74) | 49.85 (3.87) | 54.36 (3.31) | 42.18 (3.30) |
| 32 | 24.509 | α-patchoulene | 73.54 (2.31) | | 77.93 (2.61) | 61.28 (2.16) | 93.06 (2.57) | 81.71 (2.92) | 47.42 (2.00) | 67.51 (2.04) | 58.87 (2.00) | 66.87 (2.05) | 47.51 (1.59) | 68.49 (1.85) | 70.45 (1.98) | 80.27 (2.12) | 74.56 (1.96) | 84.16 (2.00) | 56.31 (1.72) | 100.00 (2.41) | 51.65 (1.60) |
| 33 | 25.264 | germacreneB | 76.42 (2.04) | | 74.88 (2.13) | 59.03 (1.76) | 93.03 (2.18) | 77.09 (2.34) | 48.84 (1.75) | 69.68 (1.79) | 59.47 (1.71) | 71.78 (1.87) | 51.36 (1.46) | 64.56 (1.48) | 72.97 (1.74) | 80.64 (1.81) | 70.28 (1.57) | 83.86 (1.69) | 79.99 (2.08) | 100.00 (2.04) | 63.21 (1.66) |
| 34 | 27.081 | (E)-nerolidol | 61.5 (15.15) | | 52.40 (13.77) | 45.88 (12.67) | 69.41 (15.02) | 36.22 (10.14) | 51.66 (17.07) | 68.6 (16.29) | 66.84 (17.78) | 72.68 (17.49) | 40.60 (10.66) | 85.12 (18.00) | 74.9 (16.54) | 59.94 (12.44) | 76.29 (15.74) | 100.00 (18.66) | 53.01 (12.74) | 64.79 (12.24) | 58.91 (14.3) |
| 35 | 27.973 | caryophyllene oxide | 16.57 (0.53) | | 10.86 (0.37) | 25.04 (0.90) | 14.94 (0.42) | 12.70 (0.46) | 19.70 (0.84) | 21.43 (0.66) | 11.03 (0.38) | 11.03 (0.34) | 36.30 (1.24) | 19.67 (0.54) | 24.46 (0.70) | 24.46 (0.66) | 19.63 (0.53) | 19.90 (0.48) | 18.30 (0.57) | 17.37 (0.43) | 18.39 (0.58) |
| 36 | 32.573 | γ-eudesmol | - | | - | - | - | - | - | - | - | - | - | - | - | - | - | - | - | - | - |
| 37 | 32.859 | eremophilene | - | | - | - | - | - | - | - | - | - | - | - | - | - | - | - | - | - | - |
| 38 | 33.482 | longifolenaldehyde | - | | - | - | - | - | - | - | - | - | - | - | - | - | - | - | - | - | - |
| 39 | 33.9 | cubenol | 70.13 (10.61) | | 61.60 (9.95) | 56.73 (9.63) | 71.17 (9.46) | 44.83 (7.71) | 56.86 (11.54) | 83.19 (12.14) | 76.94 (12.58) | 73.53 (10.88) | 44.60 (7.19) | 100.00 (12.99) | 77.10 (10.47) | 60.94 (7.77) | 72.67 (9.21) | 96.55 (11.07) | 56.35 (8.32) | 75.70 (8.79) | 69.48 (10.37) |
| 40 | 34.094 | santalol,cis,α- | 84.30 (3.81) | | 71.92 (3.46) | 50.59 (2.56) | 85.71 (3.40) | 42.50 (2.18) | 62.86 (3.81) | 94.45 (4.11) | 82.16 (4.01) | 84.36 (3.72) | 44.21 (2.13) | 98.07 (3.80) | 82.13 (3.33) | 65.64 (2.50) | 77.07 (2.91) | 100.00 (3.42) | 65.82 (2.90) | 75.60 (2.62) | 72.40 (3.22) |
| 41 | 34.631 | widdrol | - | | - | - | - | - | - | - | - | - | - | - | - | - | - | - | - | - | - |
| 42 | 35.065 | acetic acid, 3-hydroxy-6-isopropenyl-4,8a-dimethyl-1,2,3,5,6,7,8,8a-octahydronaphthalen-2-yl ester | 80.17 (0.71) | | 61.09 (0.58) | 39.22 (0.39) | 75.00 (0.58) | 42.15 (0.42) | 55.27 (0.65) | 92.99 (0.79) | 73.49 (0.70) | 84.20 (0.73) | 41.21 (0.39) | 93.94 (0.71) | 77.89 (0.62) | 57.96 (0.43) | 70.61 (0.52) | 100.00 (.67) | 58.15 (0.50) | 67.14 (0.45) | 66.22 (0.58) |
| 43 | 36.083 | 4-(2-Acetyl-5,5-dimethylcyclopent-2-enylidene)butan-2-one | | - | - | - | - | - | - | - | - | - | - | - | - | - | - | - | - | - | - |
| 44 | 36.231 | aristolene epoxide | 20.28 (0.62) | | 15.63 (0.51) | 16.29 (0.56) | 17.79 (0.48) | 12.68 (0.44) | 15.65 (0.64) | 23.61 (0.69) | 14.15 (0.47) | 22.55 (0.67) | 36.67 (1.19) | 11.54 (0.300) | 28.92 (0.79) | 27.48 (0.71) | 16.98 (0.43) | 37.85 (0.87) | 30.70 (0.91) | 23.85 (0.56) | 28.20 (0.85) |
| 45 | 37.2 | nerolidyl acetate | 67.07 (0.43) | | 59.55 (0.41) | 51.08 (0.37) | 73.40 (0.41) | 55.69 (0.40) | 57.30 (0.49) | 82.96 (0.51) | 70.06 (0.48) | 83.25 (0.52) | 37.48 (0.25) | 100.00 (0.55) | 67.71 (0.39) | 59.95 (0.32) | 74.15 (0.40) | 99.85 (0.48) | 46.19 (0.29) | 77.36 (0.38) | 69.48 (0.44) |
| 46 | 37.46 | 6-(1-hydroxymethylvinyl)-4,8a-dimethyl-3,5,6,7,8,8a-hexahydro-1H-naphthalen-2-one | 16.70 (0.32) | | 15.57 (0.31) | 20.74 (0.44) | 16.09 (0.27) | 11.91 (0.26) | 13.60 (0.34) | 23.86 (0.43) | 16.03 (0.33) | 18.47 (0.34) | 46.81 (0.94) | 13.96 (0.23) | 17.83 (0.30) | 28.23 (0.45) | 16.19 (0.26) | 27.06 (0.39) | 22.77 (0.42) | 14.93 (0.22) | 23.65 (0.44) |
| 47 | 37.946 | santalol | 57.20 (0.13) | | 49.33 (0.12) | 51.13 (0.13) | 77.09 (0.16) | 38.50 (0.10) | 47.60 (0.15) | 39.20 (0.09) | 34.97 (0.09) | 96.22 (0.22) | 36.24 (0.09) | 84.52 (0.17) | 69.88 (0.14) | 58.94 (0.11) | 50.55 (0.10) | 77.67 (0.14) | 35.20 (0.08) | 100.00 (0.18) | 64.13 (0.15) |
| 48 | 39.5 | cembrene | 15.20 (0.07) | | 12.70 (0.06) | 16.18 (0.08) | 16.13 (0.06) | 8.67 (0.05) | 12.07 (0.07) | 23.57 (0.10) | 14.65 (0.07) | 19.86 (0.09) | 33.28 (0.16) | 16.84 (0.07) | 17.11 (0.07) | 22.21 (0.09) | 13.56 (0.05) | 34.40 (0.12) | 28.88 (0.13) | 24.62 (0.09) | 29.53 (0.13) |
| Total |  |  | 75.94 (97.39) | | 69.96 (95.74) | 66.68 (95.91) | 85.32 (96.17) | 66.73 (97.33) | 55.84 (96.04) | 76.57 (94.69) | 67.35 (93.30) | 78.81 (98.80) | 66.47 (90.86) | 84.02 (92.51) | 83.63 (96.19) | 90.60 (97.90) | 86.66 (93.09) | 100.00 (97.17) | 74.24 (92.89) | 96.16 (94.62) | 75.65 (95.65) |
|  | | Chemical classes |  | |  |  |  |  |  |  |  |  |  |  |  |  |  |  |  |  |  |
|  | | Monoterpene hydrocarbons | 25.14 (3.23) | | 38.75 (5.31) | 35.17 (5.07) | 16.43 (1.85) | 41.69 (6.09) | 18.85 (3.25) | 14.60 (1.81) | 24.40 (3.38) | 16.89 (2.12) | 35.20 (4.82) | 24.72 (2.73) | 19.10 (2.20) | 36.41 (3.94) | 35.98 (3.87) | 23.37 (2.27) | 17.11 (2.14) | 34.63 (3.41) | 25.29 (3.20) |
|  | | Oxygenated monoterpenes | 66.88 (15.93) | | 58.62 (14.90) | 51.81 (13.84) | 63.56 (13.31) | 65.97 (17.87) | 42.38 (13.54) | 61.16 (14.05) | 45.74 (11.77) | 55.97 (13.03) | 56.82 (14.43) | 88.55 (18.11) | 82.52 (17.63) | 81.68 (16.39) | 100. (19.95) | 96.27 (17.38) | 67.87 (15.77) | 83.11 (15.19) | 68.54 (16.09) |
|  | | Sesquiterpene hydrocarbons | 68.04 (45.54) | | 64.16 (45.83) | 64.71 (48.58) | 84.72 (49.84) | 66.97 (50.98) | 48.17 (43.25) | 66.22 (42.74) | 56.83 (41.09) | 72.73 (47.59) | 63.94 (45.62) | 59.86 (34.4) | 70.62 (42.4) | 89.79 (50.64) | 69.34 (38.87) | 80.43 (40.79) | 71.44 (46.66) | 95.35 (48.97) | 67.05 (44.24) |
|  | | Oxygenated sesquiterpenes | 67.40 (31.16) | | 57.77 (28.5) | 51.86 (26.89) | 71.89 (29.21) | 40.51 (21.3) | 55.46 (34.39) | 77.20 (34.42) | 71.33 (35.62) | 74.51 (33.67) | 47.56 (23.43) | 90.76 (36.02) | 77.83 (32.27) | 63.24 (24.63) | 75.34 (29.17) | 100. (35.03) | 57.51 (25.94) | 70.56 (25.03) | 65.61 (29.9) |
|  | | Others | 30.62 (1.53) | | 22.57 (1.20) | 27.38 (1.53) | 44.79 (1.96) | 19.16 (1.09) | 24.22 (1.62) | 34.72 (1.67) | 26.5 (1.43) | 48.96 (2.39) | 48.15 (2.56) | 29.26 (1.25) | 37.76 (1.69) | 54.55 (2.29) | 29.25 (1.22) | 44.98 (1.70) | 48.8 (2.37) | 52.93 (2.02) | 44.89 (2.21) |

^a^ The comparative index was calculated as: comparative index (A/SI) = P(A/SI)/P(A_Max_.)*100; (comparative index (A/SI) was the comparative index of compound A in sample SI; P(A/SI) was the peak area of compound A in sample SI; P(A_Max_.) was the maximum peak area of compound A in different parts of *A. villosum* samples).

^b^ Relative content was calculated as: relative content (A/SI) = P(A/SI)/P(SI)*100%; (relative content (A/SI) was the relative content of compound A in sample SI; P(A/SI) was the peak area of compound A in sample SI; P(SI) was the total peak area of detected volatile compounds in sample SI).

- Trace or not detected.

All data was presented as average of two determinations, their relative average deviations were less than 5.3%.

**Table S1. Continued (Sample 19-36).**

| Code | RT（min） | Compounds | sample | | | | | | | | | | | | | | | | | |  |
| --- | --- | --- | --- | --- | --- | --- | --- | --- | --- | --- | --- | --- | --- | --- | --- | --- | --- | --- | --- | --- | --- |
|  |  |  | 19 | 20 | 21 | 22 | 23 | 24 | 25 | 26 | 27 | 28 | 29 | 30 | 31 | 32 | 33 | 34 | 35 | 36 | |
| 1 | 5.26 | (-)-β-pinene | 11.93 (1.55) | 6.09 (0.89) | 4.58 (0.67) | 7.24 (0.90) | 5.41 (0.78) | 13.10 (1.53) | 6.69 (0.91) | 12.39 (1.38) | 8.28 (1.21) | 5.23 (0.57) | 8.37 (1.36) | 4.52 (0.52) | 2.88 (0.46) | 10.67 (1.36) | 3.51 (0.33) | 4.25 (0.38) | 2.61 (0.42) | 3.81 (0.38) | |
| 2 | 6.117 | D-limonene | 24.60 (2.78) | 12.16 (1.55) | 16.11 (2.05) | 11.40 (1.24) | 14.81 (1.86) | 27.58 (2.80) | 8.89 (1.05) | 30.45 (2.95) | 10.93 (1.39) | 10.94 (1.04) | 33.11 (4.67) | 13.52 (1.36) | 24.87 (3.43) | 7.31 (0.81) | 17.81 (1.44) | 33.11 (2.57) | 12.87 (1.80) | 24.33 (2.10) | |
| 3 | 6.483 | trans-β-ocimene | - | - | - | - | - | - | - | - | - | - | - | - | - | - | - | - | - | - | |
| 4 | 6.786 | γ-terpinene | 78.34 (1.98) | 94.06 (2.68) | 80.51 (2.29) | 73.62 (1.78) | 67.99 (1.91) | 70.64 (1.60) | 62.36 (1.64) | 46.84 (1.01) | 70.03 (1.99) | 57.32 (1.21) | 53.90 (1.70) | 49.97 (1.13) | 53.87 (1.66) | 47.38 (1.17) | 100.00 (1.81) | 50.56 (0.88) | 88.23 (2.76) | 48.15 (0.93) | |
| 5 | 8.651 | terpinolene | 74.7 (1.12) | 93.32 (1.58) | 47.78 (0.81) | 100.00 (1.43) | 46.69 (0.77) | 86.64 (1.16) | 72.41 (1.13) | 48.19 (0.62) | 85.4 (1.43) | 44.96 (0.56) | 48.49 (0.90) | 43.77 (0.58) | 23.32 (0.43) | 22.51 (0.33) | 51.38 (0.55) | 22.63 (0.23) | 21.67 (0.40) | 24.09 (0.27) | |
| 6 | 9.095 | camphor | 10.57 (1.24) | 13.07 (1.73) | 4.04 (0.53) | 5.89 (0.66) | 13.41 (1.74) | 14.75 (1.55) | 5.45 (0.66) | 4.47 (0.45) | 5.73 (0.75) | 3.64 (0.36) | 3.81 (0.55) | 3.30 (0.34) | 2.87 (0.41) | 7.32 (0.84) | 13.51 (1.13) | 2.65 (0.21) | 3.07 (0.44) | 2.74 (0.24) | |
| 7 | 9.69 | borneol | - | - | - | - | - | - | - | - | - | - | - | - | - | - | - | - | - | - | |
| 8 | 9.998 | 4-terpineol | 36.42 (1.45) | 25.83 (1.16) | 20.53 (0.92) | 17.60 (0.67) | 43.25 (1.90) | 21.04 (0.75) | 23.64 (0.98) | 23.75 (0.81) | 23.81 (1.06) | 44.13 (1.47) | 34.82 (1.72) | 37.54 (1.33) | 21.89 (1.06) | 24.09 (0.94) | 30.11 (0.86) | 46.87 (1.28) | 28.31 (1.39) | 22.77 (0.69) | |
| 9 | 10.369 | terpineol | 20.41 (0.67) | 15.68 (0.58) | 13.15 (0.49) | 13.96 (0.44) | 44.24 (1.61) | 16.13 (0.47) | 19.84 (0.68) | 14.02 (0.39) | 40.85 (1.50) | 31.95 (0.88) | 17.96 (0.73) | 18.39 (0.54) | 22.33 (0.89) | 25.64 (0.82) | 37.3 (0.88) | 21.43 (0.48) | 15.53 (0.63) | 15.72 (0.39) | |
| 10 | 11.232 | fenchyl acetate | 94.1 (1.24) | 49.00 (0.73) | 36.83 (0.55) | 49.08 (0.62) | 38.65 (0.56) | 61.31 (0.72) | 51.96 (0.71) | 57.33 (0.64) | 41.92 (0.62) | 100.00 (1.10) | 38.64 (0.63) | 79.34 (0.93) | 45.05 (0.72) | 42.51 (0.55) | 72.18 (0.68) | 91.27 (0.82) | 51.95 (0.84) | 83.05 (0.83) | |
| 11 | 13.296 | bornyl acetate | 1.47 (0.36) | 0.97 (0.27) | 0.77 (0.21) | 1.03 (0.25) | 8.38 (2.31) | 4.65 (1.04) | 3.19 (0.82) | 1.55 (0.33) | 1.59 (0.44) | 5.02 (1.04) | 1.03 (0.32) | 4.67 (1.03) | 2.05 (0.62) | 1.33 (0.32) | 3.82 (0.68) | 4.32 (0.74) | 1.94 (0.60) | 2.50 (0.47) | |
| 12 | 13.57 | methyl 2,5-octadecadiynoate | 16.08 (0.58) | 29.44 (1.20) | 11.43 (0.47) | 45.92 (1.59) | 44.22 (1.77) | 37.88 (1.23) | 29.50 (1.11) | 48.86 (1.51) | 31.67 (1.28) | 32.52 (0.98) | 29.04 (1.31) | 30.48 (0.98) | 15.92 (0.70) | 8.65 (0.31) | 9.37 (0.24) | 14.69 (0.36) | 8.13 (0.36) | 25.09 (0.69) | |
| 13 | 14.45 | myrtenyl acetate | 24.25 (0.26) | 30.51 (0.37) | 23.6 (0.29) | 47.02 (0.48) | 27.01 (0.32) | 22.47 (0.22) | 33.92 (0.38) | 39.08 (0.36) | 22.83 (0.28) | 18.04 (0.16) | 19.84 (0.27) | 16.60 (0.16) | 15.74 (0.21) | 17.18 (0.18) | 18.52 (0.14) | 33.84 (0.25) | 14.68 (0.20) | 25.26 (0.21) | |
| 14 | 14.862 | elixene | - | - | - | - | - | - | - | - | - | - | - | - | - | - | - | - | - | - | |
| 15 | 15.302 | δ-elemene | 58.92 (0.50) | 46.47 (0.45) | 25.62 (0.24) | 31.52 (0.26) | 28.18 (0.27) | 56.12 (0.43) | 51.26 (0.45) | 43.94 (0.32) | 40.04 (0.38) | 100.00 (0.71) | 26.29 (0.28) | 91.96 (0.70) | 54.85 (0.57) | 58.05 (0.48) | 53.87 (0.33) | 57.84 (0.34) | 53.03 (0.56) | 54.68 (0.35) | |
| 16 | 17.548 | naphthalene, 1,2,3,4,4a,7-hexahydro-1,6-dimethyl-4-(1-methylethyl)- | - | - | - | - | - | - | - | - | - | - | - | - | - | - | - | - | - | - | |
| 17 | 18.222 | caryophyllene | 21.73 (8.12) | 25.04 (10.56) | 26.76 (11.27) | 30.7 (10.99) | 22.03 (9.13) | 20.05 (6.72) | 18.75 (7.29) | 32.44 (10.36) | 19.16 (8.03) | 26.94 (8.42) | 19.04 (8.86) | 24.78 (8.25) | 18.46 (8.41) | 15.12 (5.53) | 30.46 (8.15) | 35.60 (9.14) | 18.10 (8.36) | 29.53 (8.40) | |
| 18 | 19.08 | aromadendrene | - | - | - | - | - | - | - | - | - | - | - | - | - | - | - | - | - | - | |
| 19 | 19.491 | Epi-β-Santalene | - | - | - | - | - | - | - | - | - | - | - | - | - | - | - | - | - | - | |
| 20 | 19.794 | α-caryophyllene | - | - | - | - | - | - | - | - | - | - | - | - | - | - | - | - | - | - | |
| 21 | 20.006 | (Z)-β-farnesene | - | - | - | - | - | - | - | - | - | - | - | - | - | - | - | - | - | - | |
| 22 | 20.183 | allo-aromadendrene | 44.47 (4.04) | 35.37 (3.62) | 31.97 (3.27) | 59.44 (5.17) | 28.44 (2.86) | 55.74 (4.54) | 33.63 (3.18) | 50.57 (3.92) | 37.34 (3.80) | 60.69 (4.61) | 32.71 (3.70) | 56.37 (4.56) | 46.31 (5.13) | 66.07 (5.87) | 66.43 (4.32) | 62.94 (3.92) | 39.83 (4.47) | 69.09 (4.77) | |
| 23 | 20.892 | unknown | 73.09 (4.88) | 41.35 (3.11) | 46.31 (3.48) | 64.15 (4.10) | 42.57 (3.15) | 83.03 (4.96) | 43.64 (3.03) | 61.52 (3.51) | 35.67 (2.67) | 63.22 (3.53) | 45.56 (3.79) | 60.14 (3.57) | 43.68 (3.55) | 60.26 (3.93) | 88.14 (4.21) | 100.00 (4.58) | 42.21 (3.48) | 75.67 (3.84) | |
| 24 | 21.257 | γ-muurolene | - | - | - | - | - | - | - | - | - | - | - | - | - | - | - | - | - | - | |
| 25 | 21.457 | α-curcumene | - | - | - | - | - | - | - | - | - | - | - | - | - | - | - | - | - | - | |
| 26 | 21.594 | chamigrene | 48.60 (25.74) | 37.63 (22.5) | 53.10 (31.69) | 43.87 (22.26) | 46.24 (27.15) | 54.90 (26.07) | 54.67 (30.14) | 69.55 (31.49) | 42.43 (25.22) | 65.02 (28.81) | 48.47 (31.98) | 59.81 (28.23) | 41.13 (26.57) | 47.53 (24.63) | 66.51 (25.23) | 100. (36.38) | 37.89 (24.79) | 81.7 (32.94) | |
| 27 | 22.217 | α-amorphene | - | - | - | - | - | - | - | - | - | - | - | - | - | - | - | - | - | - | |
| 28 | 22.272 | β-guaiene | 67.46 (11.68) | 46.12 (9.01) | 42.02 (8.19) | 76.79 (12.73) | 37.91 (7.27) | 88.39 (13.71) | 42.06 (7.57) | 52.71 (7.80) | 40.37 (7.84) | 56.77 (8.22) | 50.03 (10.78) | 54.52 (8.41) | 47.64 (10.05) | 78.3 (13.25) | 100. (12.39) | 65.75 (7.81) | 43.82 (9.37) | 84.88 (11.18) | |
| 29 | 22.658 | cis-α-bisabolene | - | - | - | - | - | - | - | - | - | - | - | - | - | - | - | - | - | - | |
| 30 | 23.143 | β-bisabolene | 20.60 (5.06) | 24.08 (6.67) | 18.97 (5.25) | 46.66 (10.97) | 17.89 (4.87) | 25.41 (5.59) | 21.21 (5.42) | 26.69 (5.60) | 29.65 (8.17) | 27.51 (5.65) | 17.82 (5.45) | 25.47 (5.57) | 23.06 (6.91) | 26.03 (6.25) | 50.60 (8.90) | 33.37 (5.63) | 22.46 (6.81) | 21.24 (3.97) | |
| 31 | 23.931 | δ-cadinene | 55.26 (6.98) | 35.38 (5.04) | 30.75 (4.37) | 58.91 (7.12) | 28.41 (3.97) | 73.14 (8.28) | 36.70 (4.82) | 55.05 (5.94) | 35.82 (5.07) | 55.10 (5.82) | 40.65 (6.39) | 51.87 (5.83) | 41.18 (6.34) | 62.74 (7.75) | 67.94 (6.14) | 60.84 (5.27) | 36.22 (5.65) | 63.74 (6.12) | |
| 32 | 24.509 | α-patchoulene | - | - | - | - | - | - | - | - | - | - | - | - | - | - | - | - | - | - | |
| 33 | 25.264 | germacreneB | - | - | - | - | - | - | - | - | - | - | - | - | - | - | - | - | - | - | |
| 34 | 27.081 | (E)-nerolidol | - | - | - | - | - | - | - | - | - | - | - | - | - | - | - | - | - | - | |
| 35 | 27.973 | caryophyllene oxide | 44.85 (2.28) | 65.25 (3.74) | 59.45 (3.40) | 61.41 (2.98) | 57.53 (3.23) | 42.40 (1.93) | 54.70 (2.89) | 62.14 (2.69) | 76.10 (4.33) | 83.49 (3.54) | 40.30 (2.55) | 75.33 (3.40) | 49.45 (3.06) | 52.66 (2.61) | 63.62 (2.31) | 100.00 (3.48) | 50.35 (3.16) | 65.08 (2.51) | |
| 36 | 32.573 | γ-eudesmol | 8.77 (1.52) | 10.62 (2.08) | 8.73 (1.70) | 9.46 (1.57) | 8.02 (1.54) | 18.73 (2.91) | 15.44 (2.78) | 6.09 (0.90) | 9.50 (1.85) | 16.20 (2.35) | 5.27 (1.14) | 13.76 (2.12) | 13.83 (2.92) | 32.45 (5.49) | 20.95 (2.60) | 9.87 (1.17) | 13.83 (2.96) | 11.22 (1.48) | |
| 37 | 32.859 | eremophilene | 52.30 (4.38) | 50.40 (4.77) | 64.28 (6.07) | 33.34 (2.68) | 62.32 (5.79) | 38.41 (2.89) | 35.65 (3.11) | 34.32 (2.46) | 32.64 (3.07) | 66.00 (4.63) | 34.50 (3.60) | 63.68 (4.76) | 36.42 (3.72) | 50.11 (4.11) | 65.88 (3.95) | 78.66 (4.53) | 36.42 (3.77) | 53.31 (3.40) | |
| 38 | 33.482 | longifolenaldehyde | 32.08 (1.41) | 51.06 (2.53) | 58.56 (2.89) | 28.18 (1.18) | 56.51 (2.75) | 25.00 (0.98) | 55.93 (2.55) | 35.79 (1.34) | 33.14 (1.63) | 31.04 (1.14) | 26.27 (1.44) | 29.82 (1.17) | 23.76 (1.27) | 31.11 (1.33) | 51.15 (1.61) | 21.89 (0.66) | 23.76 (1.29) | 20.21 (0.67) | |
| 39 | 33.9 | cubenol | 5.18 (1.25) | 4.44 (1.20) | 7.00 (1.90) | 3.82 (0.88) | 6.66 (1.77) | 4.61 (0.99) | 10.08 (2.52) | 4.60 (0.95) | 4.34 (1.17) | 11.63 (2.34) | 2.80 (0.84) | 11.06 (2.37) | 4.06 (1.19) | 7.23 (1.70) | 7.19 (1.24) | 15.19 (2.51) | 4.06 (1.21) | 8.98 (1.64) | |
| 40 | 34.094 | santalol,cis,α- | - | - | - | - | - | - | - | - | - | - | - | - | - | - | - | - | - | - | |
| 41 | 34.631 | widdrol | - | - | - | - | - | - | - | - | - | - | - | - | - | - | - | - | - | - | |
| 42 | 35.065 | acetic acid, 3-hydroxy-6-isopropenyl-4,8a-dimethyl-1,2,3,5,6,7,8,8a-octahydronaphthalen-2-yl ester | - | - | - | - | - | - | - | - | - | - | - | - | - | - | - | - | - | - | |
| 43 | 36.083 | 4-(2-Acetyl-5,5-dimethylcyclopent-2-enylidene)butan-2-one | - | - | - | - | - | - | - | - | - | - | - | - | - | - | - | - | - | - | |
| 44 | 36.231 | aristolene epoxide | 38.94 (1.88) | 36.08 (1.97) | 47.12 (2.57) | 13.23 (0.61) | 45.36 (2.43) | 17.20 (0.75) | 34.94 (1.76) | 34.09 (1.41) | 52.64 (2.86) | 18.75 (0.76) | 15.33 (0.92) | 17.24 (0.74) | 13.84 (0.82) | 27.99 (1.32) | 36.42 (1.26) | 13.4 (0.45) | 13.84 (0.83) | 11.47 (0.42) | |
| 45 | 37.2 | nerolidyl acetate | - | - | - | - | - | - | - | - | - | - | - | - | - | - | - | - | - | - | |
| 46 | 37.46 | 6-(1-hydroxymethylvinyl)-4,8a-dimethyl-3,5,6,7,8,8a-hexahydro-1H-naphthalen-2-one | 44.24 (1.33) | 39.72 (1.34) | 67.23 (2.27) | 57.18 (1.64) | 65.45 (2.18) | 57.66 (1.55) | 77.98 (2.43) | 43.93 (1.13) | 100.00 (3.37) | 86.98 (2.18) | 49.96 (1.87) | 91.46 (2.44) | 77.35 (2.83) | 78.07 (2.29) | 53.87 (1.16) | 78.59 (1.62) | 77.35 (2.87) | 65.11 (1.49) | |
| 47 | 37.946 | santalol | - | - | - | - | - | - | - | - | - | - | - | - | - | - | - | - | - | - | |
| 48 | 39.5 | cembrene | 68.95 (0.50) | 46.22 (0.38) | 100.00 (0.82) | 54.70 (0.38) | 98.01 (0.79) | 50.36 (0.33) | 66.18 (0.50) | 82.44 (0.51) | 53.74 (0.44) | 77.46 (0.47) | 50.22 (0.46) | 74.85 (0.49) | 44.24 (0.39) | 45.22 (0.32) | 57.10 (0.30) | 98.39 (0.49) | 44.24 (0.40) | 64.45 (0.36) | |
| Total |  |  | 46.52 (94.77) | 39.89 (91.72) | 42.99 (98.67) | 48.99 (95.59) | 41.93 (94.69) | 52.39 (95.68) | 42.70 (90.52) | 52.13 (90.78) | 40.19 (91.85) | 54.31 (92.53) | 38.70 (98.2) | 50.43 (91.53) | 37.97 (94.31) | 47.43 (94.49) | 63.64 (92.82) | 68.76 (96.2) | 35.68 (89.79) | 58.53 (90.76) | |
|  | | Chemical classes |  |  |  |  |  |  |  |  |  |  |  |  |  |  |  |  |  |  | |
|  |  | Monoterpene hydrocarbons | 36.45 (7.44) | 29.13 (6.71) | 25.34 (5.82) | 27.40 (5.35) | 23.52 (5.32) | 38.77 (7.09) | 22.24 (4.72) | 34.14 (5.95) | 26.29 (6.02) | 19.81 (3.38) | 33.95 (8.63) | 19.79 (3.60) | 24.02 (5.98) | 18.39 (3.67) | 28.28 (4.13) | 29.01 (4.06) | 21.33 (5.38) | 23.67 (3.67) | |
|  |  | Oxygenated monoterpenes | 13.78 (5.22) | 11.32 (4.83) | 7.00 (2.98) | 8.60 (3.12) | 20.14 (8.45) | 14.00 (4.75) | 10.75 (4.23) | 9.21 (2.98) | 10.96 (4.65) | 15.81 (5.00) | 8.97 (4.23) | 12.85 (4.33) | 8.48 (3.91) | 9.85 (3.65) | 16.12 (4.37) | 14.57 (3.79) | 8.76 (4.10) | 9.84 (2.83) | |
|  |  | Sesquiterpene hydrocarbons | 62.54 (66.50) | 52.18 (62.62) | 58.73 (70.36) | 70.87 (72.17) | 52.02 (61.31) | 71.57 (68.22) | 56.02 (61.99) | 74.70 (67.89) | 51.62 (61.59) | 75.18 (66.86) | 53.65 (71.05) | 69.99 (66.31) | 52.21 (67.69) | 65.25 (67.86) | 91.16 (69.41) | 100.00 (73.02) | 48.55 (63.78) | 87.90 (71.14) | |
|  |  | Oxygenated sesquiterpenes | 13.15 (9.66) | 15.51 (12.86) | 17.8 (14.73) | 12.62 (8.87) | 17.08 (13.90) | 13.83 (9.11) | 19.55 (14.94) | 13.41 (8.42) | 18.45 (15.20) | 20.04 (12.31) | 9.56 (8.75) | 18.72 (12.25) | 13.50 (12.09) | 20.54 (14.76) | 19.34 (10.17) | 19.61 (9.89) | 13.56 (12.3) | 14.70 (8.22) | |
|  |  | Others | 75.25 (5.96) | 52.52 (4.69) | 53.43 (4.77) | 80.04 (6.07) | 65.06 (5.71) | 91.87 (6.52) | 56.32 (4.64) | 81.71 (5.53) | 49.45 (4.39) | 75.23 (4.98) | 56.26 (5.55) | 71.46 (5.04) | 48.14 (4.65) | 58.88 (4.56) | 83.79 (4.75) | 100.00 (5.44) | 43.35 (4.24) | 81.13 (4.89) | |

**Table S1. Continued (Sample 37-54).**

| Code | RT（min） | Compounds | sample | | | | | | | | | | | | | | | | | |
| --- | --- | --- | --- | --- | --- | --- | --- | --- | --- | --- | --- | --- | --- | --- | --- | --- | --- | --- | --- | --- |
|  |  |  | 37 | 38 | 39 | 40 | 41 | 42 | 43 | 44 | 45 | 46 | 47 | 48 | 49 | 50 | 51 | 52 | 53 | 54 |
| 1 | 5.26 | (-)-β-pinene | 53.01 (9.87) | 94.5 (11.17) | 97.36 (13.94) | 19.99 (2.62) | 21.98 (2.95) | 73.05 (13.61) | 21.37 (2.96) | 73.95 (6.89) | 33.32 (2.79) | 36.51 (5.99) | 100.00 (12.67) | 40.59 (5.48) | 51.12 (6.53) | 25.27 (3.20) | 32.34 (3.33) | 89.72 (17.96) | 22.82 (3.48) | 34.41 (6.69) |
| 2 | 6.117 | D-limonene | 42.11 (6.81) | 63.60 (6.53) | 45.5 (5.66) | 60.53 (6.89) | 26.87 (3.14) | 49.59 (8.03) | 21.88 (2.64) | 53.69 (4.35) | 44.04 (3.21) | 33.81 (4.82) | 65.17 (7.18) | 73.28 (8.60) | 60.49 (6.72) | 32.58 (3.58) | 52.36 (4.69) | 34.18 (5.95) | 56.14 (7.45) | 47.72 (8.07) |
| 3 | 6.483 | trans-β-ocimene | - | - | - | - | - | - | - | - | - | - | - | - | - | - | - | - | - | - |
| 4 | 6.786 | γ-terpinene | - | - | - | - | - | - | - | - | - | - | - | - | - | - | - | - | - | - |
| 5 | 8.651 | terpinolene | - | - | - | - | - | - | - | - | - | - | - | - | - | - | - | - | - | - |
| 6 | 9.095 | camphor | - | - | - | - | - | - | - | - | - | - | - | - | - | - | - | - | - | - |
| 7 | 9.69 | borneol | - | - | - | - | - | - | - | - | - | - | - | - | - | - | - | - | - | - |
| 8 | 9.998 | 4-terpineol | - | - | - | - | - | - | - | - | - | - | - | - | - | - | - | - | - | - |
| 9 | 10.369 | terpineol | - | - | - | - | - | - | - | - | - | - | - | - | - | - | - | - | - | - |
| 10 | 11.232 | fenchyl acetate | - | - | - | - | - | - | - | - | - | - | - | - | - | - | - | - | - | - |
| 11 | 13.296 | bornyl acetate | - | - | - | - | - | - | - | - | - | - | - | - | - | - | - | - | - | - |
| 12 | 13.57 | methyl 2,5-octadecadiynoate | 64.30 (3.32) | 28.42 (0.93) | 48.21 (1.92) | 37.69 (1.37) | 90.55 (3.38) | 24.02 (1.24) | 28.85 (1.11) | 64.65 (1.67) | 93.76 (2.18) | 35.38 (1.61) | 49.62 (1.74) | 37.31 (1.40) | 48.72 (1.73) | 27.02 (0.95) | 97.00 (2.78) | 100.00 (5.55) | 30.47 (1.29) | 82.27 (4.44) |
| 13 | 14.45 | myrtenyl acetate | 59.34 (0.91) | 42.89 (0.42) | 24.68 (0.29) | 46.27 (0.50) | 59.07 (0.66) | 21.41 (0.33) | 36.84 (0.42) | 53.63 (0.41) | 64.6 (0.45) | 85.81 (1.16) | 100.00 (1.05) | 89.56 (1.00) | 70.81 (0.75) | 40.82 (0.43) | 51.79 (0.44) | 40.31 (0.67) | 30.77 (0.39) | 80.76 (1.30) |
| 14 | 14.862 | elixene | 32.10 (3.75) | 33.47 (2.48) | 35.76 (3.22) | 49.79 (4.10) | 55.23 (4.66) | 30.24 (3.54) | 66.00 (5.75) | 89.49 (5.24) | 100.00 (5.27) | 29.96 (3.09) | 38.55 (3.07) | 40.63 (3.45) | 61.79 (4.96) | 70.78 (5.62) | 78.41 (5.08) | 31.06 (3.91) | 34.96 (3.35) | 26.48 (3.24) |
| 15 | 15.302 | δ-elemene | - | - | - | - | - | - | - | - | - | - | - | - | - | - | - | - | - | - |
| 16 | 17.548 | naphthalene, 1,2,3,4,4a,7-hexahydro-1,6-dimethyl-4-(1-methylethyl)- | - | - | - | - | - | - | - | - | - | - | - | - | - | - | - | - | - | - |
| 17 | 18.222 | caryophyllene | 40.21 (21.49) | 85.01 (28.83) | 58.84 (24.18) | 88.39 (33.23) | 76.74 (29.61) | 42.63 (22.80) | 74.30 (29.56) | 97.57 (26.08) | 100.00 (24.07) | 74.60 (35.15) | 66.59 (24.21) | 60.56 (23.48) | 61.31 (22.47) | 79.54 (28.87) | 87.48 (25.88) | 48.55 (27.88) | 65.91 (28.88) | 56.97 (31.8) |
| 18 | 19.08 | aromadendrene | 34.34 (1.44) | 44.56 (1.18) | 42.19 (1.36) | 43.47 (1.28) | 46.22 (1.40) | 35.27 (1.48) | 73.19 (2.28) | 100.00 (2.10) | 85.88 (1.62) | 34.57 (1.28) | 37.06 (1.06) | 51.49 (1.57) | 68.96 (1.98) | 69.56 (1.98) | 60.18 (1.40) | 49.24 (2.22) | 33.94 (1.17) | 38.72 (1.69) |
| 19 | 19.491 | Epi-β-Santalene | 9.95 (0.45) | 15.88 (0.46) | 12.11 (0.42) | 11.52 (0.37) | 12.68 (0.42) | 11.01 (0.50) | 21.39 (0.72) | 26.90 (0.61) | 23.15 (0.47) | 13.07 (0.52) | 14.20 (0.44) | 12.88 (0.42) | 20.67 (0.64) | 22.23 (0.68) | 23.06 (0.58) | 9.48 (0.46) | 10.14 (0.38) | 13.14 (0.62) |
| 20 | 19.794 | α-caryophyllene | 46.45 (3.38) | 92.10 (4.26) | 60.94 (3.41) | 100.00 (5.13) | 78.92 (4.15) | 41.95 (3.06) | 77.13 (4.19) | 95.86 (3.49) | 92.14 (3.02) | 91.5 (5.88) | 89.39 (4.43) | 65.68 (3.47) | 62.84 (3.14) | 81.66 (4.04) | 94.49 (3.81) | 48.27 (3.78) | 67.76 (4.05) | 59.56 (4.53) |
| 21 | 20.006 | (Z)-β-farnesene | - | - | - | - | - | - | - | - | - | - | - | - | - | - | - | - | - | - |
| 22 | 20.183 | allo-aromadendrene | 19.06 (2.47) | 33.80 (2.78) | 38.11 (3.80) | 38.45 (3.51) | 35.49 (3.33) | 18.50 (2.40) | 45.56 (4.40) | 68.36 (4.44) | 69.46 (4.06) | 23.56 (2.70) | 29.87 (2.64) | 39.51 (3.72) | 50.41 (4.49) | 49.25 (4.34) | 48.65 (3.5) | 15.95 (2.23) | 33.56 (3.57) | 17.97 (2.44) |
| 23 | 20.892 | unknown | 21.55 (2.06) | 19.31 (1.17) | 20.27 (1.49) | 17.22 (1.16) | 15.95 (1.10) | 14.33 (1.37) | 21.07 (1.50) | 33.88 (1.62) | 45.33 (1.95) | 14.56 (1.22) | 19.06 (1.24) | 27.34 (1.89) | 22.37 (1.46) | 22.88 (1.48) | 18.19 (.96) | 13.98 (1.43) | 19.59 (1.53) | 14.32 (1.43) |
| 24 | 21.257 | γ-muurolene | - | - | - | - | - | - | - | - | - | - | - | - | - | - | - | - | - | - |
| 25 | 21.457 | α-curcumene | 41.60 (8.93) | 70.22 (9.56) | 46.99 (7.75) | 42.58 (6.43) | 48.87 (7.57) | 58.87 (12.64) | 62.87 (10.05) | 70.11 (7.53) | 96.68 (9.35) | 67.15 (12.71) | 79.56 (11.62) | 63.52 (9.89) | 63.16 (9.30) | 70.38 (10.26) | 100.00 (11.88) | 33.69 (7.77) | 54.9 (9.66) | 41.76 (9.36) |
| 26 | 21.594 | chamigrene | - | - | - | - | - | - | - | - | - | - | - | - | - | - | - | - | - | - |
| 27 | 22.217 | α-amorphene | - | - | - | - | - | - | - | - | - | - | - | - | - | - | - | - | - | - |
| 28 | 22.272 | β-guaiene | - | - | - | - | - | - | - | - | - | - | - | - | - | - | - | - | - | - |
| 29 | 22.658 | cis-α-bisabolene | - | - | - | - | - | - | - | - | - | - | - | - | - | - | - | - | - | - |
| 30 | 23.143 | β-bisabolene | 7.92 (2.78) | 9.25 (2.06) | 15.34 (4.14) | 18.67 (4.61) | 13.84 (3.51) | 8.61 (3.03) | 15.40 (4.03) | 18.21 (3.2) | 28.89 (4.57) | 7.58 (2.35) | 15.15 (3.62) | 14.87 (3.79) | 15.4 (3.71) | 18.91 (4.51) | 20.40 (3.96) | 6.28 (2.37) | 16.81 (4.84) | 7.34 (2.69) |
| 31 | 23.931 | δ-cadinene | 74.94 (13.52) | 69.18 (7.93) | 32.83 (4.56) | 27.41 (3.48) | 26.20 (3.41) | 24.71 (4.46) | 31.03 (4.17) | 65.25 (5.89) | 62.41 (5.07) | 26.44 (4.21) | 42.42 (5.21) | 72.54 (9.50) | 36.74 (4.55) | 30.37 (3.72) | 27.94 (2.79) | 26.52 (5.15) | 29.85 (4.42) | 25.81 (4.87) |
| 32 | 24.509 | α-patchoulene | - | - | - | - | - | - | - | - | - | - | - | - | - | - | - | - | - | - |
| 33 | 25.264 | germacreneB | - | - | - | - | - | - | - | - | - | - | - | - | - | - | - | - | - | - |
| 34 | 27.081 | (E)-nerolidol | - | - | - | - | - | - | - | - | - | - | - | - | - | - | - | - | - | - |
| 35 | 27.973 | caryophyllene oxide | 22.20 (1.61) | 18.28 (0.84) | 17.24 (0.96) | 23.77 (1.21) | 21.44 (1.12) | 8.18 (0.59) | 13.90 (0.75) | 21.44 (0.78) | 30.64 (1.00) | 18.90 (1.21) | 27.01 (1.33) | 24.31 (1.28) | 23.54 (1.17) | 22.88 (1.13) | 29.28 (1.18) | 13.30 (1.04) | 25.02 (1.49) | 22.72 (1.72) |
| 36 | 32.573 | γ-eudesmol | - | - | - | - | - | - | - | - | - | - | - | - | - | - | - | - | - | - |
| 37 | 32.859 | eremophilene | - | - | - | - | - | - | - | - | - | - | - | - | - | - | - | - | - | - |
| 38 | 33.482 | longifolenaldehyde | 32.03 (2.01) | 44.50 (1.77) | 72.78 (3.51) | 40.57 (1.79) | 95.92 (4.34) | 100.00 (6.28) | 63.66 (2.97) | 82.34 (2.58) | 95.03 (2.69) | 26.75 (1.48) | 57.86 (2.47) | 28.11 (1.28) | 99.54 (4.28) | 41.25 (1.76) | 92.29 (3.20) | 32.86 (2.22) | 98.35 (5.06) | 26.95 (1.77) |
| 39 | 33.9 | cubenol | - | - | - | - | - | - | - | - | - | - | - | - | - | - | - | - | - | - |
| 40 | 34.094 | santalol,cis,α- | - | - | - | - | - | - | - | - | - | - | - | - | - | - | - | - | - | - |
| 41 | 34.631 | widdrol | - | - | - | - | - | - | - | - | - | - | - | - | - | - | - | - | - | - |
| 42 | 35.065 | acetic acid, 3-hydroxy-6-isopropenyl-4,8a-dimethyl-1,2,3,5,6,7,8,8a-octahydronaphthalen-2-yl ester | - | - | - | - | - | - | - | - | - | - | - | - | - | - | - | - | - | - |
| 43 | 36.083 | 4-(2-Acetyl-5,5-dimethylcyclopent-2-enylidene)butan-2-one | - | - | - | - | - | - | - | - | - | - | - | - | - | - | - | - | - | - |
| 44 | 36.231 | aristolene epoxide | 32.32 (2.24) | 53.12 (2.33) | 68.8 (3.66) | 38.03 (1.85) | 80.7 (4.03) | 94.97 (6.58) | 77.04 (3.97) | 99.32 (3.44) | 62.11 (1.94) | 31.54 (1.92) | 32.79 (1.54) | 64.22 (3.22) | 100.00 (4.75) | 33.68 (1.58) | 43.09 (1.65) | 27.00 (2.01) | 84.19 (4.78) | 29.72 (2.15) |
| 45 | 37.2 | nerolidyl acetate | - | - | - | - | - | - | - | - | - | - | - | - | - | - | - | - | - | - |
| 46 | 37.46 | 6-(1-hydroxymethylvinyl)-4,8a-dimethyl-3,5,6,7,8,8a-hexahydro-1H-naphthalen-2-one | 18.01 (0.77) | 15.48 (0.42) | 18.00 (0.59) | 13.75 (0.41) | 16.45 (0.51) | 30.53 (1.31) | 17.55 (0.56) | 22.26 (0.48) | 20.78 (0.40) | 15.37 (0.58) | 21.55 (0.63) | 22.51 (0.70) | 27.49 (0.81) | 14.95 (0.44) | 29.98 (0.71) | 16.97 (0.78) | 28.39 (1.00) | 13.93 (0.62) |
| 47 | 37.946 | santalol | - | - | - | - | - | - | - | - | - | - | - | - | - | - | - | - | - | - |
| 48 | 39.5 | cembrene | 39.52 (0.41) | 33.75 (0.22) | 29.48 (0.24) | 19.09 (0.14) | 33.65 (0.25) | 49.97 (0.52) | 32.98 (0.26) | 34.07 (0.18) | 49.32 (0.23) | 47.56 (0.44) | 41.57 (0.29) | 47.77 (0.36) | 57.49 (0.41) | 33.54 (0.24) | 58.49 (0.34) | 27.07 (0.30) | 37.11 (0.32) | 27.69 (0.30) |
| Total |  |  | 30.29 (88.23) | 46.17 (85.36) | 38.00 (85.10) | 39.08 (81.28) | 37.82 (82.02) | 32.17 (93.78) | 37.94 (82.29) | 55.57 (85.44) | 56.66 (82.09) | 34.39 (88.32) | 43.61 (86.43) | 39.99 (84.51) | 41.96 (83.84) | 39.83 (84.85) | 48.47 (84.00) | 29.92 (93.66) | 36.47 (87.10) | 29.49 (89.73) |
|  | | Chemical classes |  |  |  |  |  |  |  |  |  |  |  |  |  |  |  |  |  |  |
|  |  | Monoterpene hydrocarbons | 57.20 (16.68) | 95.62 (17.70) | 87.40 (19.60) | 46.35 (9.65) | 28.94 (6.28) | 74.15 (21.65) | 25.78 (5.60) | 77.00 (11.86) | 45.70 (6.63) | 42.07 (10.82) | 100.00 (19.85) | 66.57 (14.09) | 66.20 (13.25) | 34.21 (7.3) | 49.70 (8.63) | 76.24 (23.9) | 45.72 (10.93) | 48.45 (14.76) |
|  |  | Oxygenated monoterpenes | 1.69 (0.91) | 1.22 (0.42) | 0.70 (0.29) | 1.31 (.51) | 1.68 (0.68) | 0.61 (0.33) | 1.05 (0.42) | 1.52 (.44) | 1.84 (.49) | 2.44 (1.16) | 2.84 (1.05) | 2.55 (1.00) | 2.01 (0.75) | 1.16 (0.46) | 1.47 (0.47) | 1.15 (0.67) | 0.87 (0.39) | 2.30 (1.30) |
|  |  | Sesquiterpene hydrocarbons | 38.30 (58.22) | 61.72 (59.55) | 45.21 (52.85) | 58.09 (63.06) | 52.89 (59.86) | 35.43 (53.92) | 57.55 (65.15) | 77.02 (61.81) | 83.97 (63.50) | 50.63 (67.88) | 54.41 (56.29) | 53.75 (59.29) | 52.97 (55.24) | 62.01 (68.94) | 69.95 (63.28) | 34.12 (55.76) | 48.38 (60.31) | 38.56 (61.24) |
|  |  | Oxygenated sesquiterpenes | 6.31 (6.63) | 8.05 (5.37) | 10.81 (8.73) | 7.13 (5.35) | 13.20 (10.32) | 14.04 (14.76) | 10.56 (8.25) | 13.85 (7.68) | 12.73 (6.65) | 5.61 (5.19) | 8.36 (5.98) | 8.51 (6.48) | 15.28 (11.01) | 6.87 (5.28) | 11.60 (7.25) | 5.35 (6.04) | 14.31 (12.32) | 5.71 (6.26) |
|  |  | Others | 51.15 (5.79) | 32.35 (2.32) | 41.79 (3.64) | 33.47 (2.71) | 57.87 (4.88) | 27.63 (3.13) | 33.95 (2.86) | 61.19 (3.66) | 85.52 (4.82) | 32.79 (3.27) | 42.53 (3.28) | 44.46 (3.65) | 46.37 (3.60) | 34.69 (2.87) | 64.99 (4.38) | 59.92 (7.29) | 33.83 (3.14) | 52.17 (6.17) |

**Table S1. Continued (Sample 55-72).**

| Code | RT（min） | Compounds | sample | | | | | | | | | | | | | | | | | |
| --- | --- | --- | --- | --- | --- | --- | --- | --- | --- | --- | --- | --- | --- | --- | --- | --- | --- | --- | --- | --- |
|  |  |  | 55 | 56 | 57 | 58 | 59 | 60 | 61 | 62 | 63 | 64 | 65 | 66 | 67 | 68 | 69 | 70 | 71 | 72 |
| 1 | 5.26 | (-)-β-pinene | 37.37 (4.71) | 15.34 (2.98) | 11.58 (2.46) | 12.66 (3.20) | 11.96 (3.03) | 10.80 (3.42) | 14.24 (3.43) | 36.06 (4.41) | 42.82 (4.33) | 14.89 (2.55) | 13.11 (1.93) | 14.22 (2.95) | 7.31 (2.05) | 6.12 (1.88) | 34.56 (3.83) | 11.01 (2.44) | 5.39 (1.88) | 15.84 (2.48) |
| 2 | 6.117 | D-limonene | 100.00 (10.95) | 28.58 (4.82) | 49.08 (9.07) | 59.75 (13.12) | 42.98 (9.47) | 35.92 (9.88) | 45.76 (9.59) | 90.66 (9.64) | 76.94 (6.76) | 55.42 (8.23) | 49.82 (6.36) | 45.32 (8.17) | 32.82 (8.01) | 24.83 (6.64) | 81.65 (7.86) | 45.19 (8.72) | 31.97 (9.69) | 60.80 (8.26) |
| 3 | 6.483 | trans-β-ocimene | - | - | - | - | - | - | - | - | - | - | - | - | - | - | - | - | - | - |
| 4 | 6.786 | γ-terpinene | - | - | - | - | - | - | - | - | - | - | - | - | - | - | - | - | - | - |
| 5 | 8.651 | terpinolene | - | - | - | - | - | - | - | - | - | - | - | - | - | - | - | - | - | - |
| 6 | 9.095 | camphor | 89.18 (10.10) | 41.56 (7.25) | 25.99 (4.97) | 43.86 (9.96) | 35.25 (8.03) | 40.73 (11.59) | 38.96 (8.44) | 87.2 (9.59) | 83.96 (7.63) | 49.58 (7.61) | 50.9 (6.72) | 42.33 (7.89) | 39.53 (9.97) | 18.29 (5.06) | 69.85 (6.95) | 39.66 (7.91) | 23.63 (7.41) | 60.16 (8.45) |
| 7 | 9.69 | borneol | - | - | - | - | - | - | - | - | - | - | - | - | - | - | - | - | - | - |
| 8 | 9.998 | 4-terpineol | 83.78 (3.22) | 63.02 (3.73) | 100.00 (6.49) | 83.10 (6.40) | 37.83 (2.92) | 48.85 (4.72) | 63.49 (4.67) | 67.29 (2.51) | 34.96 (1.08) | 82.32 (4.29) | 70.74 (3.17) | 52.09 (3.29) | 65.39 (5.60) | 40.04 (3.76) | 75.21 (2.54) | 60.76 (4.11) | 54.53 (5.8) | 74.86 (3.57) |
| 9 | 10.369 | terpineol | 70.57 (2.24) | 50.06 (2.45) | 51.45 (2.76) | 61.48 (3.91) | 64.77 (4.13) | 65.58 (5.23) | 61.84 (3.75) | 86.23 (2.66) | 100.00 (2.55) | 49.33 (2.12) | 66.10 (2.45) | 69.90 (3.65) | 52.53 (3.71) | 47.51 (3.68) | 62.29 (1.74) | 53.36 (2.98) | 34.34 (3.02) | 54.4 (2.14) |
| 10 | 11.232 | fenchyl acetate | 74.93 (0.95) | 46.91 (0.92) | 56.09 (1.20) | 34.06 (0.87) | 29.73 (0.76) | 25.93 (0.83) | 34.49 (0.84) | 48.19 (0.60) | 48.21 (0.49) | 40.90 (0.71) | 59.07 (0.88) | 47.31 (0.99) | 28.90 (0.82) | 29.16 (0.91) | 50.92 (0.57) | 42.67 (0.96) | 14.71 (0.52) | 65.43 (1.03) |
| 11 | 13.296 | bornyl acetate | - | - | - | - | - | - | - | - | - | - | - | - | - | - | - | - | - | - |
| 12 | 13.57 | methyl 2,5-octadecadiynoate | 20.41 (0.71) | 6.56 (0.35) | 13.87 (0.82) | 41.33 (2.90) | 10.98 (0.77) | 27.6 (2.43) | 29.64 (1.98) | 30.39 (1.03) | 8.48 (0.24) | 38.82 (1.84) | 13.52 (0.55) | 5.14 (0.30) | 17.50 (1.36) | 5.94 (0.51) | 12.42 (0.38) | 27.11 (1.67) | 17.42 (1.69) | 17.24 (0.75) |
| 13 | 14.45 | myrtenyl acetate | - | - | - | - | - | - | - | - | - | - | - | - | - | - | - | - | - | - |
| 14 | 14.862 | elixene | - | - | - | - | - | - | - | - | - | - | - | - | - | - | - | - | - | - |
| 15 | 15.302 | δ-elemene | - | - | - | - | - | - | - | - | - | - | - | - | - | - | - | - | - | - |
| 16 | 17.548 | naphthalene, 1,2,3,4,4a,7-hexahydro-1,6-dimethyl-4-(1-methylethyl)- | - | - | - | - | - | - | - | - | - | - | - | - | - | - | - | - | - | - |
| 17 | 18.222 | caryophyllene | 1.25 (0.45) | 1.96 (1.09) | 2.11 (1.29) | 1.07 (0.77) | 2.67 (1.94) | 1.07 (0.97) | 1.16 (0.80) | 2.45 (0.86) | 4.15 (1.20) | 3.48 (1.71) | 1.32 (0.56) | 1.37 (0.81) | 1.23 (0.99) | 1.50 (1.32) | 3.28 (1.04) | 1.20 (0.76) | 0.74 (0.74) | 1.31 (0.59) |
| 18 | 19.08 | aromadendrene | - | - | - | - | - | - | - | - | - | - | - | - | - | - | - | - | - | - |
| 19 | 19.491 | Epi-β-Santalene | - | - | - | - | - | - | - | - | - | - | - | - | - | - | - | - | - | - |
| 20 | 19.794 | α-caryophyllene | - | - | - | - | - | - | - | - | - | - | - | - | - | - | - | - | - | - |
| 21 | 20.006 | (Z)-β-farnesene | - | - | - | - | - | - | - | - | - | - | - | - | - | - | - | - | - | - |
| 22 | 20.183 | allo-aromadendrene | 76.3 (6.70) | 68.20 (9.23) | 53.3 (7.90) | 39.76 (7.00) | 36.26 (6.41) | 25.84 (5.70) | 41.54 (6.98) | 74.63 (6.37) | 85.14 (6.00) | 70.46 (8.40) | 100.00 (10.25) | 61.33 (8.87) | 50.52 (9.89) | 50.45 (10.83) | 91.26 (7.04) | 69.78 (10.80) | 40.70 (9.90) | 66.96 (7.29) |
| 23 | 20.892 | unknown | 27.08 (1.75) | 19.83 (1.97) | 18.96 (2.07) | 51.55 (6.67) | 11.30 (1.47) | 3.75 (0.61) | 11.38 (1.40) | 40.32 (2.53) | 54.19 (2.81) | 27.7 (2.43) | 13.66 (1.03) | 9.13 (0.97) | 7.50 (1.08) | 3.67 (0.58) | 21.85 (1.24) | 6.23 (0.71) | 6.18 (1.10) | 23.80 (1.90) |
| 24 | 21.257 | γ-muurolene | - | - | - | - | - | - | - | - | - | - | - | - | - | - | - | - | - | - |
| 25 | 21.457 | α-curcumene | 48.71 (7.07) | 43.68 (9.77) | 34.81 (8.53) | 17.56 (5.11) | 25.24 (7.37) | 15.22 (5.55) | 30.46 (8.46) | 56.46 (7.96) | 83.16 (9.69) | 53.42 (10.52) | 69.73 (11.81) | 39.16 (9.36) | 25.68 (8.31) | 26.53 (9.41) | 60.73 (7.75) | 45.92 (11.75) | 23.62 (9.49) | 51.60 (9.29) |
| 26 | 21.594 | chamigrene | - | - | - | - | - | - | - | - | - | - | - | - | - | - | - | - | - | - |
| 27 | 22.217 | α-amorphene | - | - | - | - | - | - | - | - | - | - | - | - | - | - | - | - | - | - |
| 28 | 22.272 | β-guaiene | 28.18 (4.72) | 24.46 (6.31) | 27.65 (7.82) | 3.58 (1.20) | 23.12 (7.79) | 11.76 (4.95) | 25.99 (8.32) | 61.43 (9.99) | 49.38 (6.63) | 43.51 (9.88) | 37.06 (7.24) | 28.39 (7.82) | 14.58 (5.44) | 16.21 (6.63) | 58.91 (8.67) | 27.48 (8.10) | 10.89 (5.05) | 26.99 (5.60) |
| 29 | 22.658 | cis-α-bisabolene | - | - | - | - | - | - | - | - | - | - | - | - | - | - | - | - | - | - |
| 30 | 23.143 | β-bisabolene | 10.29 (2.45) | 4.50 (1.65) | 7.03 (2.82) | 11.40 (5.43) | 6.93 (3.31) | 4.22 (2.52) | 5.36 (2.44) | 17.12 (3.95) | 53.30 (10.16) | 11.73 (3.78) | 6.50 (1.80) | 6.20 (2.42) | 3.15 (1.67) | 4.77 (2.77) | 25.16 (5.25) | 2.96 (1.24) | 0.69 (0.45) | 4.86 (1.43) |
| 31 | 23.931 | δ-cadinene | 60.06 (7.34) | 42.47 (7.99) | 49.89 (10.29) | 6.08 (1.49) | 27.4 (6.73) | 16.71 (5.13) | 35.07 (8.19) | 65.88 (7.81) | 100.00 (9.80) | 53.49 (8.86) | 69.73 (9.93) | 44.13 (8.87) | 27.51 (7.48) | 25.96 (7.75) | 91.95 (9.87) | 44.42 (9.56) | 27.82 (9.40) | 58.46 (8.85) |
| 32 | 24.509 | α-patchoulene | - | - | - | - | - | - | - | - | - | - | - | - | - | - | - | - | - | - |
| 33 | 25.264 | germacreneB | - | - | - | - | - | - | - | - | - | - | - | - | - | - | - | - | - | - |
| 34 | 27.081 | (E)-nerolidol | - | - | - | - | - | - | - | - | - | - | - | - | - | - | - | - | - | - |
| 35 | 27.973 | caryophyllene oxide | 56.88 (2.79) | 46.66 (3.53) | 28.84 (2.39) | 85.84 (8.45) | 15.39 (1.52) | 21.78 (2.69) | 15.59 (1.46) | 46.04 (2.19) | 36.73 (1.45) | 14.38 (.96) | 32.05 (1.83) | 32.36 (2.61) | 27.20 (2.97) | 32.22 (3.86) | 75.06 (3.24) | 20.98 (1.81) | 19.90 (2.70) | 37.69 (2.29) |
| 36 | 32.573 | γ-eudesmol | 83.02 (13.9) | 50.42 (13.00) | 36.03 (10.18) | 28.04 (9.42) | 40.53 (13.65) | 34.67 (14.59) | 37.98 (12.16) | 68.6 (11.16) | 90.51 (12.16) | 42.44 (9.64) | 78.81 (15.4) | 50.14 (13.82) | 32.88 (12.26) | 33.68 (13.78) | 100. (14.72) | 47.22 (13.93) | 28.41 (13.17) | 68.27 (14.18) |
| 37 | 32.859 | eremophilene | 100.00 (8.11) | 69.20 (8.64) | 60.45 (8.28) | 26.13 (4.25) | 30.86 (5.03) | 27.82 (5.67) | 28.36 (4.40) | 70.96 (5.59) | 86.62 (5.64) | 44.20 (4.86) | 63.74 (6.03) | 51.73 (6.90) | 25.84 (4.67) | 27.03 (5.36) | 87.99 (6.27) | 36.69 (5.24) | 22.49 (5.05) | 69.27 (6.97) |
| 38 | 33.482 | longifolenaldehyde | - | - | - | - | - | - | - | - | - | - | - | - | - | - | - | - | - | - |
| 39 | 33.9 | cubenol | - | - | - | - | - | - | - | - | - | - | - | - | - | - | - | - | - | - |
| 40 | 34.094 | santalol,cis,α- | - | - | - | - | - | - | - | - | - | - | - | - | - | - | - | - | - | - |
| 41 | 34.631 | widdrol | 76.93 (0.93) | 42.26 (0.79) | 16.10 (0.33) | 89.54 (2.17) | 39.91 (0.97) | 33.96 (1.03) | 27.53 (0.64) | 69.26 (0.81) | 23.89 (0.23) | 35.89 (0.59) | 89.09 (1.26) | 44.95 (0.90) | 23.80 (0.64) | 34.34 (1.02) | 100.00 (1.06) | 44.46 (0.95) | 15.39 (0.52) | 72.24 (1.08) |
| 42 | 35.065 | acetic acid, 3-hydroxy-6-isopropenyl-4,8a-dimethyl-1,2,3,5,6,7,8,8a-octahydronaphthalen-2-yl ester | - | - | - | - | - | - | - | - | - | - | - | - | - | - | - | - | - | - |
| 43 | 36.083 | 4-(2-Acetyl-5,5-dimethylcyclopent-2-enylidene)butan-2-one | 93.17 (2.90) | 68.61 (3.29) | 34.55 (1.81) | 19.04 (1.19) | 61.48 (3.85) | 40.18 (3.14) | 53.26 (3.17) | 86.95 (2.63) | 94.76 (2.36) | 36.08 (1.52) | 100.00 (3.63) | 76.54 (3.92) | 35.84 (2.48) | 44.2 (3.36) | 23.03 (0.63) | 57.59 (3.15) | 18.21 (1.57) | 97.29 (3.75) |
| 44 | 36.231 | aristolene epoxide | - | - | - | - | - | - | - | - | - | - | - | - | - | - | - | - | - | - |
| 45 | 37.2 | nerolidyl acetate | - | - | - | - | - | - | - | - | - | - | - | - | - | - | - | - | - | - |
| 46 | 37.46 | 6-(1-hydroxymethylvinyl)-4,8a-dimethyl-3,5,6,7,8,8a-hexahydro-1H-naphthalen-2-one | 36.06 (1.05) | 32.26 (1.44) | 22.46 (1.10) | 23.24 (1.35) | 18.49 (1.08) | 15.57 (1.14) | 9.35 (0.52) | 14.28 (0.40) | 14.35 (0.33) | 38.12 (1.50) | 33.01 (1.12) | 25.87 (1.24) | 23.99 (1.55) | 40.07 (2.84) | 49.58 (1.26) | 29.03 (1.48) | 18.43 (1.48) | 35.33 (1.27) |
| 47 | 37.946 | santalol | - | - | - | - | - | - | - | - | - | - | - | - | - | - | - | - | - | - |
| 48 | 39.5 | cembrene | 28.60 (0.20) | 13.91 (0.15) | 12.20 (0.15) | 5.84 (0.08) | 7.43 (0.11) | 8.13 (0.14) | 22.58 (0.30) | 15.78 (0.11) | 5.73 (0.03) | 19.07 (0.18) | 19.58 (0.16) | 12.78 (0.15) | 11.64 (0.18) | 6.33 (0.11) | 24.40 (0.15) | 18.19 (0.23) | 9.12 (0.18) | 35.40 (0.31) |
| Total |  |  | 47.31 (93.26) | 30.09 (91.33) | 27.87 (92.72) | 24.02 (94.96) | 22.79 (90.33) | 18.56 (91.93) | 24.39 (91.95) | 48.48 (92.79) | 57.90 (91.56) | 34.48 (92.19) | 40.93 (94.10) | 29.56 (95.88) | 20.76 (91.13) | 19.11 (92.04) | 53.15 (92.06) | 28.37 (98.51) | 16.64 (90.80) | 37.43 (91.48) |
|  | | Chemical classes |  |  |  |  |  |  |  |  |  |  |  |  |  |  |  |  |  |  |
|  |  | Monoterpene hydrocarbons | 79.34 (15.66) | 25.66 (7.80) | 34.63 (11.54) | 41.23 (16.32) | 31.49 (12.5) | 26.82 (13.30) | 34.48 (13.02) | 73.32 (14.05) | 70.02 (11.09) | 40.26 (10.78) | 36.01 (8.29) | 34.22 (11.11) | 22.88 (10.06) | 17.68 (8.53) | 67.37 (11.68) | 32.10 (11.16) | 21.18 (11.57) | 43.85 (10.73) |
|  |  | Oxygenated monoterpenes | 45.09 (16.51) | 25.44 (14.34) | 24.94 (15.41) | 28.80 (21.14) | 21.52 (15.85) | 24.31 (22.37) | 25.27 (17.70) | 43.18 (15.35) | 39.98 (11.74) | 29.67 (14.73) | 30.95 (13.22) | 26.26 (15.82) | 24.64 (20.10) | 14.99 (13.41) | 36.67 (11.80) | 24.75 (15.96) | 16.52 (16.74) | 33.45 (15.19) |
|  |  | Sesquiterpene hydrocarbons | 35.81 (36.85) | 28.20 (44.67) | 27.02 (46.92) | 12.24 (25.26) | 18.64 (38.58) | 11.79 (30.49) | 20.12 (39.59) | 42.57 (42.53) | 59.51 (49.12) | 34.41 (48.02) | 39.68 (47.62) | 26.61 (45.06) | 16.78 (38.44) | 17.53 (44.06) | 50.77 (45.9) | 26.18 (47.45) | 14.08 (40.08) | 31.38 (40.02) |
|  |  | Oxygenated sesquiterpenes | 26.28 (18.68) | 17.15 (18.76) | 11.68 (14.00) | 15.01 (21.39) | 12.05 (17.22) | 10.89 (19.45) | 10.88 (14.78) | 21.11 (14.57) | 24.86 (14.17) | 13.16 (12.69) | 23.66 (19.61) | 15.88 (18.56) | 11.01 (17.43) | 12.38 (21.5) | 32.48 (20.28) | 14.52 (18.18) | 9.09 (17.87) | 21.37 (18.82) |
|  |  | Others | 72.59 (5.56) | 48.83 (5.76) | 37.46 (4.84) | 70.57 (10.84) | 40.17 (6.19) | 32.82 (6.32) | 46.81 (6.86) | 84.59 (6.29) | 88.52 (5.44) | 57.46 (5.97) | 60.07 (5.37) | 42.28 (5.33) | 29.93 (5.11) | 24.33 (4.55) | 35.67 (2.40) | 42.67 (5.76) | 21.39 (4.54) | 70.67 (6.71) |
